# Chemical rescue of mutant proteins in living *Saccharomyces cerevisiae* cells by naturally occurring small molecules

**DOI:** 10.1093/g3journal/jkab252

**Published:** 2021-07-17

**Authors:** Daniel S Hassell, Marc G Steingesser, Ashley S Denney, Courtney R Johnson, Michael A McMurray

**Affiliations:** Department of Cell and Developmental Biology, University of Colorado Anschutz Medical Campus, Aurora, CO 80045, USA

**Keywords:** chemical biology, yeast genetics, mutant, drug action, chaperone

## Abstract

Intracellular proteins function in a complex milieu wherein small molecules influence protein folding and act as essential cofactors for enzymatic reactions. Thus protein function depends not only on amino acid sequence but also on the concentrations of such molecules, which are subject to wide variation between organisms, metabolic states, and environmental conditions. We previously found evidence that exogenous guanidine reverses the phenotypes of specific budding yeast septin mutants by binding to a WT septin at the former site of an Arg side chain that was lost during fungal evolution. Here, we used a combination of targeted and unbiased approaches to look for other cases of “chemical rescue” by naturally occurring small molecules. We report *in vivo* rescue of hundreds of *Saccharomyces cerevisiae* mutants representing a variety of genes, including likely examples of Arg or Lys side chain replacement by the guanidinium ion. Failed rescue of targeted mutants highlight features required for rescue, as well as key differences between the *in vitro* and *in vivo* environments. Some non-Arg mutants rescued by guanidine likely result from “off-target” effects on specific cellular processes in WT cells. Molecules isosteric to guanidine and known to influence protein folding had a range of effects, from essentially none for urea, to rescue of a few mutants by DMSO. Strikingly, the osmolyte trimethylamine-*N*-oxide rescued ∼20% of the mutants we tested, likely reflecting combinations of direct and indirect effects on mutant protein function. Our findings illustrate the potential of natural small molecules as therapeutic interventions and drivers of evolution.

## Introduction

Physicochemical variability in the environment drives selection for traits that allow organisms to adapt to prevailing conditions with minimal energetic cost. Temperature, hydrostatic pressure, and the concentrations of intracellular molecules strongly influence protein structure and, consequently, function. Whereas protein sequences in extremophiles provide clear signatures of adaptation to temperature, pressure, intracellular pH, and salinity (Brininger *et al.* 2018), many proteins also depend on the presence of other small molecule ligands as folding or enzymatic cofactors that confer changes in tertiary structure or catalytic activity, respectively. Thus, the amino acid sequence of a protein is not sufficient to confer proper folding and function in all environments; likewise, how changes in protein sequence affect function depends on the physicochemical environment.

Researchers commonly exploit “conditional” mutant alleles as powerful tools to manipulate gene function in living cells. Temperature-sensitive (TS) mutants typically harbor one or more missense mutations that shift the folding equilibrium such that the mutant protein functions at least somewhat normally at the “permissive” temperature but loses function at the “nonpermissive”/“restrictive” temperature. A conceptually similar approach involves substituting key amino acids for which the side chains are suspected or known to make critical intra- or inter-molecular contacts, and attempting to restore function by adding small molecules that bind at the molecular vacancy created by the mutation and restore the missing contacts. There are numerous instances in which this approach, often called “chemical rescue,” “chemical complementation,” or the “bump-and-hole tactic,” has been successful (reviewed in Marletta 2006; Peracchi 2008; Bishop and Chen 2009; Makhlynets *et al.* 2015; Islam 2018). However, we are aware of only a handful of cases in which chemical rescue of a mutant protein has been demonstrated *in vivo* using simple, common small molecules (as opposed to complex molecules tailored to a specific mutant protein).

Indole, which resembles the Trp side chain, can be produced intracellularly by tryptophanase-expressing bacteria, including *Escherichia* *coli* (Li and Young 2013). The addition of exogenous indole can rescue the function of multiple Trp-to-Gly mutant proteins created in the lab, using both structure-guided design or unbiased genetic techniques, and both *in vitro* and in living *E. coli* cells (Xia *et al.* 2013; Lopez and Anderson 2015). Thus in principle indole expands the functional sequence space available to these proteins, though it is not known whether intracellular indole concentrations are high enough for long enough to allow any organism to naturally explore that space in the context of evolution. While indole is commonly used as a scaffold to design anti-cancer drugs (Wan *et al.* 2019), Trp missense mutations are relatively rare as causes of human genetic diseases, limiting the potential for indole as a treatment via “chemical rescue.” Within a published list of single-missense mutations linked to human disease, Trp substitutions account for just 1.8% (52 of 2960) (Sahni *et al.* 2015).

By contrast, due to the abundance of mutation-prone CpG dinucleotides in Arg-encoding codons, Arg is the most frequently mutated residue in human disease (Cooper and Youssoufian 1988), representing 18% (538 of 2960) of disease-linked single-missense mutations (Sahni *et al.* 2015). In fact, the first example of *in vivo* chemical rescue involved a mutant of the protein kinase Csk in which a key Arg residue was mutated to Ala (Lowry *et al.* 2002). This study followed earlier *in vitro* studies of the same mutant in which the authors found that the activity of the mutant enzyme could be partially restored by the addition of guanidine hydrochloride (GdnHCl) or, with even greater effect, imidazole (Williams *et al.* 2000). GdnHCl dissociates to produce the guanidinium ion (Gdm), which mimics the guanidino group at the end of an Arg side chain and had been used successfully in many *in vitro* chemical rescue experiments of Arg-mutant enzymes. It was a surprise that imidazole, which resembles a His side chain and had been used previously to rescue His-mutant enzymes, also rescued Csk(R318A). Cellular indications of restored Csk activity were observed within 15 min after mouse embryonic fibroblasts expressing the Csk(R318A) enzyme were exposed to 50 mM imidazole, though the extent of rescue was estimated as about 5% of WT (Williams *et al.* 2000). Recognizing that the Arg in question is highly conserved among protein kinases in the same family as Csk, the same group went on to mutate the equivalent Arg in the protein kinase Src and show that imidazole could also rescue Src(R388A), this time to nearly 50% of WT activity using only 2 mM imidazole (Qiao *et al.* 2006).

While free imidazole is not commonly found in nature, the choice of this molecule as a rescue agent has been touted as advantageous relative to guanidine, which is often considered to be toxic (Marletta 2006). Likely for this reason, GdnHCl had not (to our knowledge) been tested for its effectiveness in chemical rescue of Arg-mutant proteins *in vivo*. However, there is abundant evidence that GdnHCl can specifically alter protein function without grossly disrupting cellular function. For decades, researchers have added 3–5 mM GdnHCl to the growth medium of the budding yeast *Saccharomyces cerevisiae* to inhibit the function of the disaggregase protein Hsp104, a nonessential protein required for the propagation of most prions (Tuite *et al.* 1981; Ferreira *et al.* 2001; Jung and Masison 2001). Gdm binds in the ATP-binding pocket of this AAA+-family ATPase (Grimminger *et al.* 2004). Notably, ≥5 mM GdnHCl cures yeast cells of the prion [*ISP*^+^] in an Hsp104-independent manner, and inhibits growth specifically of [*ISP*^+^] cells (Volkov *et al.* 2002), providing evidence of additional, Hsp104-independent effects and reinforcing the idea that GdnHCl is well tolerated by WT cells. Studies in cultured mammalian cells showed that exogenous GdnHCl blocks replication of enteroviruses (Rightsel *et al.* 1961; Loddo *et al.* 1962), with no adverse cellular effects up to 10 mM (Loddo *et al.* 1962). Subsequent work pointed to the viral 2C protein as the direct target (Pfister and Wimmer 1999). (That 2C and Hsp104 are both AAA+ ATPases may be a coincidence.) Gdm also binds in the pores of voltage-gated potassium channels and promotes acetylcholine release (Kalia and Swartz 2011), representing the likely mechanism of action in the treatment of Lambert-Eaton Myasthenic Syndrome and botulism, for which GdnHCl is approved by the Food and Drug Administration of the United States of America. When combined with another drug with a distinct target, low-dose GdnHCl is effective as a treatment of Lambert-Eaton Myasthenic Syndrome, with few side effects (Oh *et al.* 1997). Thus, far from being nonspecifically toxic, at low concentrations GdnHCl is innocuous or even beneficial, curing yeast cells of some prions and mammalian cells of some viruses, and treating some human disorders.

We were inspired to investigate the potential of GdnHCl for chemical rescue in living cells by our prior, serendipitous discovery that GdnHCl reverses the TS phenotypes of *S. cerevisiae* cells carrying mutations in the *CDC10* gene (Johnson *et al.* 2020). *CDC10* encodes a subunit of the essential yeast septin protein complex. Our data indicate that, rather than binding to mutant Cdc10 molecules, Gdm binds to WT molecules of another septin protein in the same complex, at the former site of an Arg side chain that was “lost” during fungal evolution. In many yeast species, loss of the Arg and of GTPase activity by the same septin constrained the kinds of septin hetero-oligomers that are able to assemble in those cells (Johnson *et al.* 2020). Specifically, we think that Gdm alters the conformation of the homodimerization interface that encompasses the GTP-binding pocket in a way that promotes bypass of Cdc10 subunits during septin complex assembly (Johnson *et al.* 2020). Restoring the “missing” Arg residue was insufficient to recapitulate the effect of GdnHCl, but it blocked the ability of GdnHCl to rescue, consistent with a model in which multiple amino acid changes during evolution disfavored Cdc10 bypass, and Gdm acts as a “chemical chaperone” to influence folding of a single septin protein in ways that cannot be predicted by protein sequence alone. Nonetheless, our findings lent further support to the idea that GdnHCl might be able to restore function to an Arg-mutant protein in living cells.

Guanidine itself (Makhatadze and Privalov 1992) and other small molecules isosteric with guanidine, including urea (Makhatadze and Privalov 1992) and DMSO (Arakawa *et al.* 2007), are also able to interact with proteins rather nonspecifically to alter their conformations, driving complete unfolding at sufficiently high concentrations. The effects of urea are particularly relevant *in vivo*. Some marine animals accumulate intracellular urea at high concentrations (∼0.4 M), the denaturing effects of which they counteract by also accumulating methylamine compounds, including trimethylamine *N*-oxide (TMAO) (Yancey and Somero 1979). Deep-sea creatures also accumulate TMAO to counteract protein-unfolding effects of hydrostatic pressure (Yancey *et al.* 2004). TMAO, like DMSO, is also used by some organisms in anaerobic conditions as an alternative electron receptor for respiration (McCrindle *et al.* 2005; Carey *et al.* 2018). Exogenously added TMAO has further been found to restore function to three otherwise misfolded mutant proteins in living *S. cerevisiae* and *E. coli* cells (Singh *et al.* 2007; Bandyopadhyay *et al.* 2012) and one in cultured mammalian cells (Brown *et al.* 1997). Thus intracellular TMAO and other naturally occurring compounds with similar effects on protein folding may allow the organisms that produce them to explore a larger protein sequence space (De Los Rios and Goloubinoff 2012; Bandyopadhyay *et al.* 2012). The recent discoveries that multiple bacterial species evolved ways to sense and export or detoxify intracellular Gdm (Breaker *et al.* 2017) raise the possibility that Gdm could also influence protein function and evolution in natural settings. However, we are unaware of any thorough comparison of the effects of naturally occurring small molecules on mutant protein function in living cells.

In this study, we tested the ability of GdnHCl to provide chemical rescue to a variety of Arg-mutant proteins in living cells. We also performed an unbiased search for cases of chemical rescue by GdnHCl using collections of TS mutants and then, based on our findings, examined the potential for rescue by urea, DMSO, and TMAO. Our findings document what are to our knowledge the first examples of *bona fide* GdnHCl chemical rescue *in vivo*, and provide a number of molecular insights into the “on-target” and “off-target” effects of GdnHCl, as well as mechanistic comparisons with other naturally occurring small molecules on mutant protein function.

## Materials and methods

### Microbial strains and plasmids

We screened two collections of TS mutants. Screening methods are provided below. The “Hieter collection” has the following genotype for each haploid TS mutant: *MAT***a** *ura3Δ0 leu2Δ0 his3Δ1 can1Δ::LEU2-P_MFA1_::HIS3 yfeg-ts::URA3*, where “*yfeg-ts*” is “your favorite essential gene,” of which 600 are represented (Kofoed *et al.* 2015). The allele at *MET15* was either *met15Δ0* or *MET15*^+^, and at *LYS2* was either *lys2Δ0* or *LYS2^+^*, but this information is not available for all mutants. From this collection, a separate stock of the *pga1-ts* mutant was made and assigned the inventory number H06474. The “Boone collection” that we screened comprises 684 mutants representing 444 essential genes, based on the parent strain BY4741 (*MAT***a** *his3Δ1 leu2Δ0 met15Δ0 ura3Δ0*) with each TS allele integrated using a *kanMX* marker 3’ of the stop codon (Li *et al.* 2011). Supplementary Table S1 is an updated copy of a previously published table from the Boone lab (Li *et al.* 2011) that lists the mutants in the version of the collection we screened along with the temperature(s) at which the TS phenotype is manifested and, where known, the mutations in the TS allele. The following are yeast strains not in these collections. S288C is *MAT*α *SUC2 ho mal mel gal2 CUP1 flo1 flo8-1 hap1* (Mortimer and Johnston 1986). BY4741 is derived from S288C and is *MAT***a** *his3Δ1 leu2Δ0 met15Δ0 ura3Δ0* (Brachmann *et al.* 1998a). The *erg6Δ::kanMX* derivative from the BY4741 deletion collection (Winzeler *et al.* 1999; Giaever *et al.* 2002) is named H06534 in our lab collection and was confirmed by cycloheximide sensitivity (Parsons *et al.* 2004). The *cdc10(D182N) erg6Δ* strain H06537 was made by crossing H06534 with the *cdc10(D182N)* strain DDY2334, a gift from David Drubin (University of California Berkeley), sporulating the resulting diploid, and dissecting tetrads. Other strains from the deletion collections were confirmed by other phenotypes: the *arg3Δ::kanMX* derivative of BY4741 (strain H06538) was confirmed by arginine auxotrophy, and the *asn1Δ::kanMX* derivative of BY4741 and *asn2Δ::kanMX* derivative of BY4742 (*MAT*α *his3Δ1 leu2Δ0 lys2Δ0 ura3Δ0*, Brachmann *et al.* 1998a) were confirmed by the asparagine auxotrophy of H06544, the haploid *asn1Δ::kanMX asn2Δ::kanMX* strain created by mating those two mutants, sporulating the resulting diploid, and dissecting tetrads. Strains AKY19 (*pga1-1*) and KSY182 (*pga1-3*) are *MET15* derivatives of BY4741 and have *LEU2* integrated at the *PGA1* locus (Sato *et al.* 2007). The p53 reporter strain RBy33 is *MAT***a** *his3Δ200 leu2Δ1 lys2Δ202 trp1Δ63 1cUAS53::URA3* (Brachmann *et al.* 1996). The original *mcm3-1* mutant strain Rc61-3c is *MAT*α *his4Δ34 leu2-3,112 ura3-52 mcm3(G246E)* (Lei *et al.* 2002). The *ssaΔ* strains ECY487 (*SSA1*) and ECY567 (*ssa1-45BKD*) are both *MAT*α*leu2-3,112 his3-11,3-15 trp1Δ1 ura3-52 ssa2::LEU2 ssa3::TRP1 ssa4::LYS2* (Kim *et al.* 1998). The *act1(R312A)* strain H06780 is a derivative of BY471 made by co-transformation of two PCR products, one made using template plasmid YCpU-act1(R312A) (see below) and primers Act1FW (5’CCTCGTGCTGTCTTCCCATC) and act1(R312A)-A2 (5’GTGCACTCTCAGTACAATCTGCTCTGATGCCGCATAGTTAAGCCAGCCCCGACACTTATATTACATACATTTGCG), and the other made using template plasmid pRS406 (Christianson *et al.* 1992) and primers 5_pRS (5’GTGTCGGGGCTGGCTTAACTATGCGGCATCAGAGCAGATTGTACTGAGAGTGCAC) and act1(R312A)-A4 (5’ATGACTTTACAGTTATTGGTTCAATTTAGCGGTATAAACATTATCCTTGGCTGTGCGGTATTTCACACCG).

The *E. coli* strains used were DH5α [*fhuA2 lac(del)U169 phoA glnV44 Φ80' lacZ(del)M15 gyrA96 recA1 relA1 endA1 thi-1 hsdR17*] and RS302 (alias AMA1004), which is based on strain M182 (Casadaban and Cohen 1980) with the following modifications: *hsdR- del(ara, leu) trpC9830 leuB6 ara+ Δ(laclPOZ)C29 lacY^+^*.

All yeast transformations were performed using the Frozen-EZ Yeast Transformation II Kit (#T2001, Zymo Research). To integrate the *ura3(R235A)* allele, the Cas9-encoding plasmid [Cas9-NAT (Zhang *et al.* 2014], a gift from Yong-Su Jin, Addgene plasmid # 64329; http://n2t.net/addgene:64329; RRID: Addgene_64329) was transformed into S288C and selected with nourseothricin. A geneblock repair template (Supplementary Figure S1) containing the R235A mutation or a recoded version of WT *URA3* was amplified by PCR using primers ura3_GBfw (5’TCCATGGAGGGCACAGTTAA) and ura3_GBre (5’CACCCTCTACCTTAGCATCC) and the PCR product was co-transformed into S288C cells carrying the Cas9 plasmid with a gRNA-encoding plasmid, gRNA-ura-HYB (Zhang *et al.* 2014) (a gift from Yong-Su Jin, Addgene plasmid #64330; http://n2t.net/addgene:64330; RRID: Addgene_64330). Transformants arising on YPD medium with nourseothricin and hygromycin B were screened by isolation of genomic DNA, PCR of the *URA3* coding sequence, and DNA sequencing, resulting in the strains H06537 [*ura3(R235A)*] and H06700 (*URA3*).

The appropriate Arg mutations were introduced into plasmids pPact1-lacZ, YCpU-Pgal-ARG3, pGALOTC, YCpU-Pgal-ASN1, or pKFW29 via site-directed mutagenesis performed by Keyclone Technologies (San Marcos, CA), resulting in plasmids G00602 [pPact1–lacZ(R599A)], G00612 [YCpU-Pgal-arg3(R69G)], G00639 [hOTC(R92G)], G00648 [YCpU-Pgal-asn1(R344A)], and G00672 [YCpU-act1(R312A)], respectively. To introduce the R249S mutant of p53 into a yeast plasmid, pLT11, a low-copy *HIS3*-marked plasmid encoding p53(V272M) under control of the yeast *ADH1* promoter (Dearth *et al.* 2007), was digested with *Sna*BI and *Eco*RI to excise the region including codons 249 and 272, and co-transformed into yeast cells with uncut plasmid pCMV-Neo-Bam p53(R249S), a gift from Bert Vogelstein (Addgene plasmid #16438; http://n2t.net/addgene:16438; RRID: Addgene_16438) (Baker *et al.* 1990). WT p53 in the same vector backbone was created by digestion with *Sna*BI and *Stu*I of the low-copy *HIS3*-marked plasmid pTW456 (Dearth *et al.* 2007) and co-transformation into yeast cells with pMAM38b, a *LEU2*-marked plasmid encoding WT p53. (The WT p53 sequence in pMAM38 was derived from plasmid p663/LR78, Gaglia and Lahav 2014). Transformants arising on medium lacking histidine were rescued to *E. coli* and confirmed by sequencing with appropriate primers. The R249S plasmid, called pMAM71, was subjected to site-directed mutagenesis to introduce the H168A mutation by Keyclone Technologies, generating plasmid pMAM70, and then pMAM70 was mutagenized in the same way to introduce the T123A mutation and create pMAM113. Deletion of the mitochondrial import sequence of HsOTC was performed by co-transforming into WT (BY4741) yeast cells *Bam*HI-digested pGALOTC and a double-stranded DNA molecule made by annealing oligonucleotides newhOTCdelNfw (5′-TCCTTTACACAATTAAAAGAAGATGCTGTTTAATCTGAGGGACCTTCTCACTCTAAAAAACTTTACCGGAGAAGAAATTA) and hOTCdelNre (5′-TAATTTCTTCTCCGGTAAAGTTTTTTAGAGTGAGAAGGTCCCTCAGATTAAACAGCATCTTCTTTTAATTGTGTAAAGGA). Transformants arising on medium lacking uracil were rescued to *E. coli* and confirmed by PCR and sequencing with appropriate primers. The resulting plasmid is called G00796. pRS426 (Christianson *et al.* 1992) was used as a *URA3*-marked empty vector.

### Media and additives

*S. cerevisiae* rich growth medium was YPD (1% yeast extract (#Y20020, Research Products International, Mount Prospect, IL), 2% peptone (#P20241, RPI), 2% dextrose [#G32045, RPI)]. Supplementation with extra nutrients to test for defects in nutrient uptake was done by adding Leu, His, and uracil to 0.1, 0.05, and 0.1 mg/mL, respectively. Synthetic complete growth medium was based on YC (0.1 g/L Arg, Leu, Lys, Thr, Trp, and uracil; 0.05 g/L Asp, His, Ile, Met, Phe, Pro, Ser, Tyr, and Val; 0.01 g/L adenine; 1.7 g/L Yeast Nitrogen Base without amino acids or ammonium sulfate; 5 g/L ammonium sulfate; 2% dextrose or galactose) with individual components (from Sigma Aldrich, St. Louis, MO, or RPI) eliminated as appropriate for plasmid selection. For solid media, agar (#A20030, RPI Corp.) was added to 2%. For counterselection against *URA3*, 5-fluoro-orotic acid monohydrate (#F5050, United States Biological, Salem, MA) was added to modified YC medium with 50 µg/mL uracil and 0.1% FOA, for experiments with p53 mutants, or 20 µg/mL uracil and 400 µg/mL FOA for experiments with Ura3 mutants. Nourseothricin (#N5375-74, US Biological) was added to YPD at 100 µg/mL. Hygromycin B (#H-270-1, Gold Biotechnology) was added to YPD at 200 µg/mL. GdnHCl (#G4505, Sigma Aldrich, St. Louis, MO or #G49000, RPI), aminoguanidine HCl (#sc-202931, Santa Cruz Biotechnology, Santa Cruz, CA), N-ethylguanidine HCl (#sc-269833, Santa Cruz), metformin hydrochloride (#M2009, TCI Chemicals), urea (#1610730, Bio-Rad Laboratories, Inc.), TMAO (#AAA1491614, Alfa Aesar), were dissolved in water. MG132 (#10012628, Cayman Chemical Company) was dissolved in ethanol to make a 75 mM stock solution. Medium for MG132 contained, per L: 20 g agar, 20 g dextrose, 0.017 g Yeast Nitrogen Base without amino acids or ammonium sulfate, 1 g Pro, 0.1 g Leu, 0.1 g uracil, 0.05 g His, 0.05 g Met, 0.03 g SDS. Rich medium for *E. coli* was LB (per L: 5 g yeast extract, 10 g tryptone, 10 g sodium chloride), supplemented with carbenicillin or ampicillin and, as appropriate, X-gal (Fisher BioReagents #bp1615-1) from a stock dissolved in dimethylformamide (for experiments with strain RS302) or DMSO (for experiments with DH5α).

### Kinetic β-gal assay

We grew 5-mL LB+ampicillin cultures with DH5α carrying pACT1-lacZ or its R599A derivative to mid-log phase at 37˚, then added 8 µl of X-gal (20 mg/mL in DMSO) and 5 µl of chloramphenicol (Sigma-Aldrich # C0378, 34 mg/mL dissolved in ethanol). After time intervals of 0, 10, 20, 50, 100, 120, or 150 min, a 500-µl aliquot of cells was removed and pelleted, then resuspended in 250 µl of 50 mM Tris-HCl, 10 mM EDTA, pH 8.0, 100 µg/mL RNase A, followed by addition of 250 µl 200 mM sodium hydroxide, 1% SDS to lyse the cells. 100-µl aliquots of this lysate were transferred to the wells of a flat-bottom 96-well microplate. Absorbance at 615 nm was measured with a BioTek Cytation 3 plate reader. Plasmid was isolated from the remainder of the original 5-mL culture of the R599A culture using standard methods and digested with *Pvu*I-HF (New England Biolabs #R3151) according to manufacturer’s instructions. Digested DNA was separated by 1% agarose gel electrophoresis in Tris-acetate EDTA buffer and visualized with ethidium bromide.

### Screening the TS collections

We obtained the Hieter collection as a set of seven plates with colonies on solid medium from the lab of Mark Johnston (University of Colorado Anschutz Medical Campus). A ROTOR bench-top robot (Singer Instruments, Somerset, England) was used to duplicate the colonies to YPD. After incubation at RT, 531 of the 604 mutants formed colonies. These were then pinned by the robot in 384-colony format to two YPD plates and two YPD plates containing 3 mM GdnHCl. One of each type was incubated at RT and the other at 37˚. Growth was scored after 6 days incubation. Unfortunately, photos of these plates were lost, so we do not know the identity of the 73 mutants that did not grow even on YPD at RT. Candidate mutants showing rescue or sensitivity to GdnHCl were streaked to new plates for re-testing.

For GdnHCl rescue, as with the Hieter collection, seven plates of the Boone collection in 96-well format, this time in liquid form, were first robotically transferred to YPD at RT. The resulting colonies were condensed to 384-well format on four YPD plates, and this was done in duplicate for each of three conditions: RT without GdnHCl, 37˚ without GdnHCl, and 37˚ with 3 mM GdnHCl. After 5 days at the indicated temperature, plates were screened visually to compare colony sizes. We note that a single mutant from the Boone collection that we scored as being rescued by GdnHCl, *erg26-1*, is also represented in the Hieter collection, albeit in a slightly different genetic background. Why it was not identified in the Hieter collection screen is unclear. For urea, DMSO, and TMAO rescue, the colonies arising on YPD after transfer from liquid stocks were pinned in quadruplicate for each of four conditions: YPD at RT and 37˚, and YPD with the selected concentration of experimental small molecule (10 mM urea, 5% DMSO, or 0.5 M TMAO) at RT and 37˚. After 3 days at the indicated temperature, plates were screened visually to compare colony sizes.

### Sequence alignments

Multiple protein sequence alignments were performed using the COBALT tool at the NCBI server (https://www.ncbi.nlm.nih.gov/tools/cobalt/) or, for sequences of yeast species not available via NCBI, using the “Fungal Alignment” function at the Yeast Genome Database (http://yeastgenome.org). In some cases, sequences obtained via the Yeast Genome Database were aligned with other sequences via COBALT.

### DNA sequencing

Genomic DNA from strains *pga1-ts* (H06474), “*cdc20-1*” (the strain found in the well of the Boone TS collection where the *cdc20-1* was supposed to be), *cdc20-2*, *cdc20-3*, *stu1-5* (H06573), the strain with WT “recoded” *URA3* (H06700), or the *ura3(R235A)* strain (H06701) was isolated as described previously (Johnson *et al.* 2015) and used as template in a Phusion (New England Biolabs, Ipswich, MA) or Taq DNA Polymerase (New England Biolabs, Ipswich, MA) PCR reaction with primers flanking to or within the coding sequences, according to the polymerase manufacturer’s instructions. These were: 5’PGA1fw (5′-TGTTGACCCTTGGTATTCGC), 3’PGA1re (5′-CCAAAGGGGGCTCAGGCTTC), cdc20_fwd (5′-ATCAGGCCTAGGACTAATCTGCCTTGC), cdc20_rev (5′-CTTTAACATGTGATGAGTTATATCTTGATG), STU1midre (5’GATAACTCATCAGTCAAGTCG), STU1-Nmidfw (5’GAATTTGTTGGCACCGTTCC), STU1-Nmidre (5’CGCTGGGTCTTTACTGCTC), 5'STU1fw (5’GCTGGCAATATAAACACAAGT), 5’URA3fw (5’GGCTGTGGTTTCAGGGTCCAT), 3'URA3re (5’GTCATTATAGAAATCATTACG). Following treatment with alkaline phosphatase (#EF0651) and Exonuclease I (#EN0581, Thermo Scientific Fermentas, Waltham, MA), the purified PCR product was directly sequenced at the sequencing facility of the Barbara Davis Center for Childhood Diabetes or Quintara Biosciences. Additional primers used for sequencing were: midPGA1fw (5′-GTCTTGTCTGACTCGTATCC), midPGA1re (5′-CTGACTCATTGCTTCTCCCG), midURA3re (5’TGCATTCGTAATGTCTGCCC), midURA3fw (5’AAATTGCAGTACTCTGCGGG).

## Results

### *In vivo* chemical rescue by guanidine hydrochloride of a subset of Arg-mutant enzymes

To ask if GdnHCl can rescue the function of Arg-mutant enzymes in living cells, we first tested a number of enzymes for which single Arg-to-Gly or Arg-to-Ala substitutions were known to reduce enzyme activity *in vitro* in a manner that could be rescued to some extent by the addition of GdnHCl. Arg at position 599 of *E. coli* β-gal makes intramolecular contacts that position the active site in an “open” conformation with high affinity for substrates (Dugdale *et al.* 2010). *In vitro*, binding of β-gal(R599A) to *ortho*- or *para-*nitrophenyl-β-D-galactoside (*o*NPG or *p*NPG) is reduced ∼10-fold relative to WT, and the kinetics of *o*NPG catalysis are reduced ∼five-fold but can be improved ∼two-fold (to ∼2.5-fold less than WT) by the addition of 100–150 mM GdnHCl (Dugdale *et al.* 2010). However, we found no obvious difference in the color of colonies produced by *E. coli* cells mutated for the endogenous *lacZ* gene and expressing WT β-gal or β-gal(R599A) from plasmids on solid medium containing X-gal (5-bromo-4-chloro-3-indoyl-β-d-galactopyranoside; neither *o*NPG nor *p*NPG are suitable for *in vivo* analysis; Supplementary Figure S2A). The R599A mutation also had no discernable effect on the kinetics of *in vivo* X-gal cleavage in liquid culture (Supplementary Figure S2B). To rule out a functional contribution by the N-terminally truncated β-gal allele encoded at the genomic locus (Ullmann *et al.* 1967), we introduced the plasmids into a strain carrying a complete deletion of *lacZ*. X-gal cleavage by β-gal(R599A) was also indistinguishable from WT in this strain (Supplementary Figure S1C). Thus, without a discernable phenotype β-gal(R599A) could not be tested for GdnHCl rescue *in vivo*.

**Figure 1:**
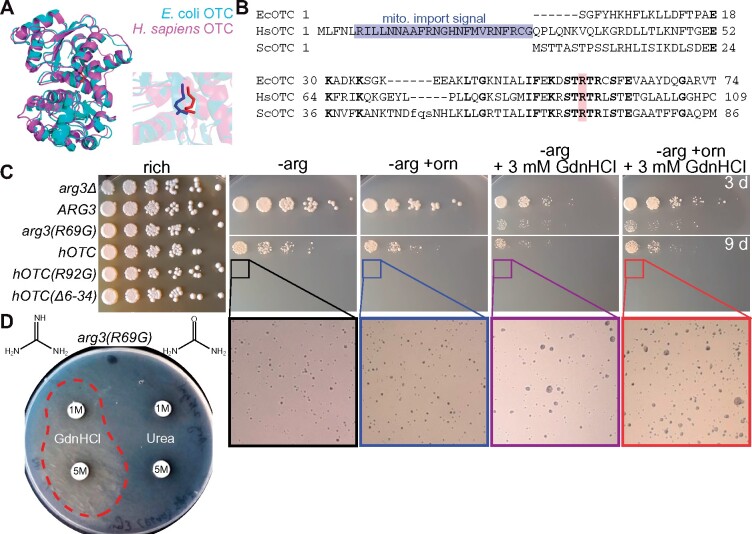
Chemical rescue by GdnHCl of Arg-mutant ornithine transcarbamylase (OTC) in living yeast cells. (A) Superimposition of crystal structures of human and *E. coli* OTC. The zoomed-in image shows the locations of the side chains of Arg57 from the bacterial enzyme (blue) and Arg92 from the human enzyme. (B) Sequence alignment of portions of the OTC homologs from *E. coli* (EcOTC), human (HsOTC), and *S. cerevisiae* (ScOTC). Bold residues are identical. Blue highlights the leader sequence that directs import of HsOTC into mitochondria. Pink highlights the position of Arg57 in EcOTC, Arg92 in HsOTC, and Arg69 in ScOTC. (C) Dilution series of cells of the indicated genotypes on rich (YPD) or synthetic medium lacking arginine (“-arg”) and thus selective for OTC function. The carbon source in these plates was 2% galactose to induce OTC expression. Where indicated, ornithine (“+orn”) and/or GdnHCl were added to final concentration of 100 µg/mL or 3 mM, respectively, to attempt to improve HsOTC function. Selective plates were incubated at 30˚ for the indicated number of days (“3 d” or “9 d”) prior to imaging; YPD plate was incubated for 3 days. The strain was H06538 carrying plasmids pRS316 (“*arg3Δ*”), YCpU-Pgal-ASN1 (“*ASN1*”), G00648 [“*arg3(R69G)*”], pGALOTC (“*hOTC*”), G00639 [“*hOTC(R92G)*”], or G00796 (*“hOTCΔ6-34*”). (D) A lawn of H06538 cells carrying plasmid G00648 was plated to media selective for OTC function and a sterile paper disc soaked with GdnHCl or urea at the indicated concentrations was placed on the lawn. The plate was then imaged after 6 days incubation at 30˚.

Mutating Arg57 in the active site of *E. coli* ornithine transcarbamylase (EcOTC) drastically reduces activity *in vitro* but the addition of GdnHCl restores it to 10% of WT (Rynkiewicz and Seaton 1996). Human OTC (HsOTC) is 38% identical to EcOTC, solved structures of the two proteins are superimposable, and the Arg57 equivalent is found at position 92 (Figure 1, A and B). Mutations in HsOTC cause ornithine transcarbamylase deficiency (OTCD), the most frequent inherited defect in the urea cycle (∼1 in 14,000 live births), characterized by hyperammonemia and a requirement for dietary Arg (Yamaguchi *et al.* 2006). Among known HsOTC mutations linked to OTCD, Arg92 has been found in a number of cases substituted to other amino acids, including Gly (Yamaguchi *et al.* 2006), indicating that HsOTC(R92G) is likely a loss-of-function allele *in vivo*. In *S. cerevisiae*, OTC is encoded by the *ARG3* gene, so named because loss-of-function mutants cannot synthesize Arg (Crabeel *et al.* 1981). The Arg3 protein (which we also refer to as “ScOTC”) is 33% identical to EcOTC and is able to complement the Arg auxotrophy of an *E. coli argF* mutant (Crabeel *et al.* 1981). Arg69 in ScOTC corresponds to Arg57 in EcOTC (Figure 1B). A plasmid-encoded copy of *ARG3*, but not *arg3(R69G)*, conferred Arg prototrophy to *arg3Δ* cells (Figure 1C). Adding 3 mM GdnHCl, a concentration that rescues *cdc10* mutants (Johnson *et al.* 2020), partially rescued ScOTC(R69G) function (Figure 1C), as did placing a disc of GdnHCl-soaked paper onto a lawn of cells (Figure 1D). Notably, an equivalent experiment with urea, which provided weak *in vitro* rescue of EcOTC(R57G) (Rynkiewicz and Seaton 1996), revealed no such effect *in vivo* (Figure 1D). WT HsOTC, but not HsOTC(R92G), partially complemented the Arg auxotrophy of *arg3* yeast (Figure 1C). The addition of 3 mM GdnHCl barely rescued HsOTC(R92G), visualized as slightly larger microcolonies after extended incubation (Figure 1C). HsOTC is normally mitochondrial but import of WT HsOTC into yeast mitochondria is inefficient, and some HsOTC remains stuck in the mitochondrial membrane (Cheng *et al.* 1987). To retain HsOTC in the cytosol, where ScOTC normally functions, we removed the 32-residue N-terminal import sequence (Figure 1B), but rather than improving HsOTC function, this alteration eliminated it (Figure 1C). We interpret these data as evidence of partial chemical rescue by GdnHCl of Arg-mutant OTC in living cells.

**Figure 2:**
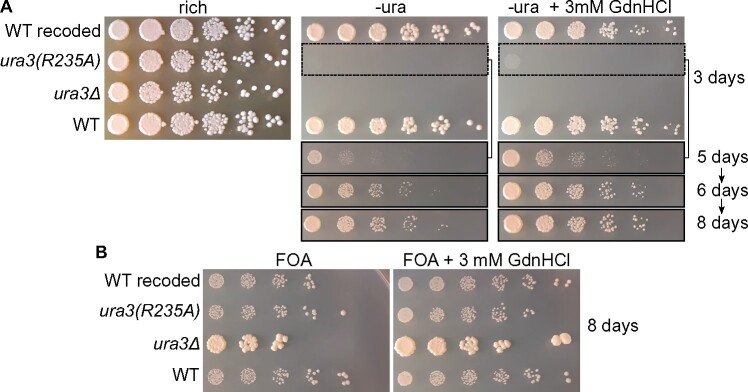
*In vivo* chemical rescue by GdnHCl of Ura3(R235A). (A) Dilution series of cells of the indicated genotypes on rich (YPD) or synthetic medium lacking uracil and thus selective for Ura3 function. Plates were incubated at 30˚ for the indicated number of days prior to imaging. Strains were: “WT,” S288C; “WT recoded,” H06700; “*ura3(R253A)*,” H06701; “*ura3Δ*,” BY4741. (B) As in (A) but on medium with 20 µg/mL uracil and 400 µg/mL FOA, which is converted by Ura3 to a toxic product that inhibits colony growth.

Due to an exceptionally high affinity for a transition state of its substrate, orotidine-5'-phosphate decarboxylase (ODCase) displays one of the largest rate enhancements (>10^17^-fold) of any known enzyme (Radzicka and Wolfenden 1995). The guanidino group in the side chain of Arg 235 of the *S. cerevisiae* ODCase, Ura3, contacts the substrate in its transition state (Miller *et al.* 2000). *In vitro*, Ura3(R235A) decarboxylates orotidine-5'-phosphate ∼15-fold more slowly than does WT Ura3, but the addition of GdnHCl at 100 mM restores activity to ∼70% of WT (Barnett *et al.* 2010). To look for rescue by GdnHCl *in vivo*, we introduced the R235A substitution into the *URA3* locus of otherwise WT cells. A guide RNA targeted Cas9 cleavage in the protein-coding sequence, which was repaired by homologous recombination with a synthetic double-stranded DNA molecule encoding WT Ura3 or Ura3(R235A) harboring synonymous substitutions within the sequences between the site of cleavage and codon 235 (Supplementary Figure S1). Consistent with the decreased ODCase activity of Ura3(R235A) *in vitro* (Barnett *et al.* 2010), *ura3(R235A)* cells formed very small colonies on medium lacking uracil (Figure 2A). These cells proliferated slightly faster when 3 mM GdnHCl was added to the media (Figure 2A), indicating weak rescue of ODCase activity. We also looked for rescue of Ura3(R235A) using 5-fluoro-orotic acid (FOA), which selects against Ura3 activity (Boeke *et al.* 1984). The threshold of Ura3 activity that allows growth in the absence of uracil is distinct from the threshold that inhibits colony growth on FOA (Gottschling *et al.* 1990). However, 3 mM GdnHCl had no discernable effect on colony growth by *ura3(R235A)* cells, which was indistinguishable from that of cells with WT Ura3 activity, and much slower than *ura3Δ* cells (Figure 2B). We conclude from these data that GdnHCl is able to rescue the function of Ura3(R235A) in living yeast cells, but to a very minor extent. Thus for two Arg-mutant enzymes with functional defects *in vivo*, GdnHCl provided partial but detectable rescue.

### Failure of GdnHCl to rescue Arg-mutant asparagine synthetase or a “rationally designed” mutant allele of p53

**Figure 3:**
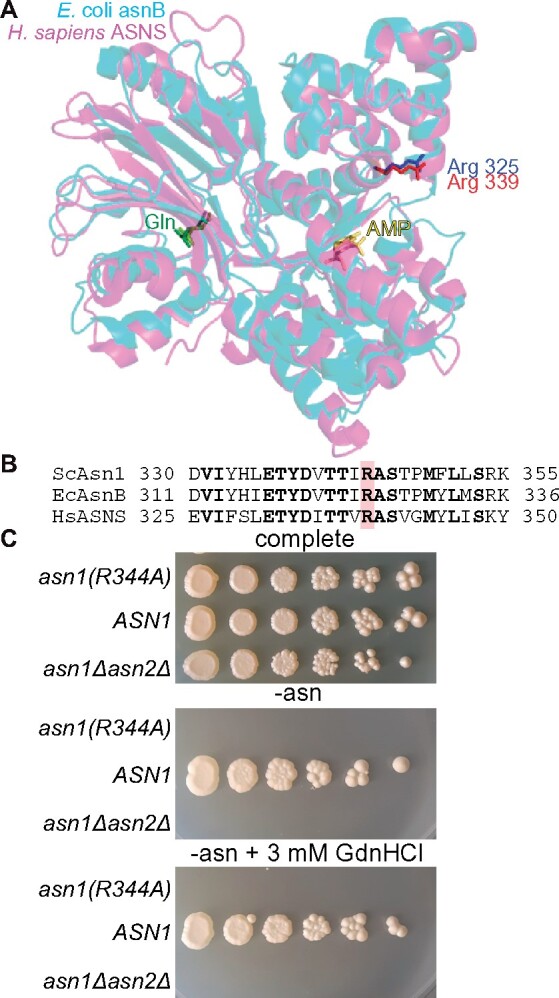
No evidence of chemical rescue of Asn1(R344A) by GdnHCl in living yeast cells. (A) Overlay of crystal structures of EcAsnB (PDB 1CT9) and HsASNS (PDB 6GQ3) showing the locations of EcAsnB Arg 325 and HsASNS Arg 339 and two bound substrates, glutamine (“Gln”) and AMP. (B) Sequence alignment of a region of asparagine synthase from *S. cerevisiae* (“ScAsn1”), *E. coli* (“EcAsnB”), and *H. sapiens* (“HsASNS”). The Arg residue corresponding to EcAsnB Arg 325 is highlighted in red. (C) Dilution series of cells of the indicated genotypes on complete (but lacking uracil) or synthetic medium lacking asparagine (and uracil) and thus selective for Asn1 function (and the *URA3*-marked plasmid). Plates were incubated at 30˚ for 6 days prior to imaging. Strain was H06544 carrying empty vector plasmid pRS426 (“*asn1Δasn2Δ*”), YCpU-Pgal-ASN1 (“*ASN1*”), or YCpU-Pgal-asn1(R344A) [“*asn1(R344A)*”].

Arg325 lies near one active site of asparagine synthetase B (Figure 3A). Mutation to Ala in the *E. coli* enzyme abolishes activity *in vitro* but 50 mM GdnHCl restores activity to ∼15% of WT (Boehlein *et al.* 1997). Mutation of the equivalent Arg in the human homolog (Figure 3, A and B) causes Asparagine Synthetase Deficiency (Sun *et al.* 2017). This Arg-mutant enzyme was thus a good candidate for chemical rescue *in vivo*. The redundant *S. cerevisiae* paralogs Asn1 and Asn2 are 47% identical to *E. coli* asparagine synthetase B. Deletion of both genes renders yeast cells auxotrophic for asparagine (Dang *et al.* 1996). We introduced the mutation R344A, corresponding to the *E. coli* mutation (Figure 3B), into Asn1 encoded on a plasmid and under control of the *GAL1/10* promoter, and looked for growth by an *asn1Δ asn2Δ* strain on media containing galactose and lacking asparagine. Only WT Asn1 allowed asparagine prototrophy, regardless of the presence of 3 mM GdnHCl in the medium (Figure 3C). We interpret these data as evidence that the Asn1(R344A) protein is not amenable to rescue *in vivo* by GdnHCl, at least to an extent that is detectable by our approach.

**Figure 4:**
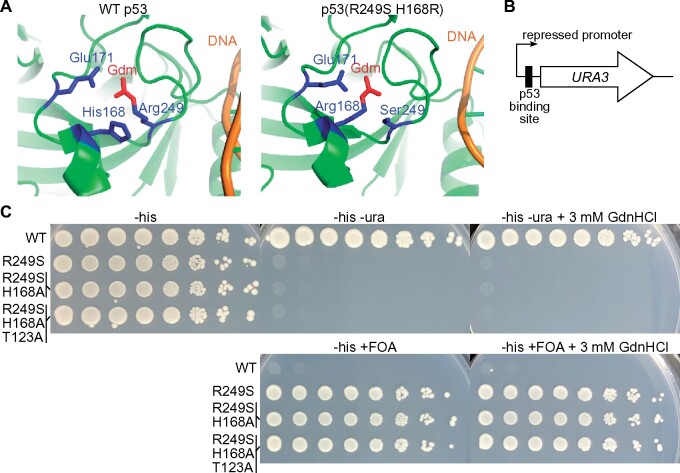
GdnHCl fails to rescue p53(R249S). (A) Crystal structures of the DNA-binding domain of WT p53 (PDB 2AC0) and p53(R249S H168R) (PDB 3D0A) showing the locations of relevant side chains and, in particular, the guanidino group (“Gdm”) occupying the same position in both proteins. Glu171 is shown because in both cases it forms a salt bridge with the guanidino group. (B) Schematic of yeast reporter system. (C) Dilution series of cells of the reporter strain (RBy33) carrying *HIS3*-marked plasmids (“WT,” pMAM44; “R249S,” pMAM71; “R249S H168A,” pMAM70; “R249S H168A T123A,” and pMAM113) were plated on synthetic medium lacking uracil (“-ura”) or containing 50 µg/mL uracil and 0.1% FOA and incubated at 30˚ prior to imaging.

We next looked for an Arg-mutant protein that would be good candidate for *in vivo* chemical rescue by GdnHCl but had not been previously tested *in vitro*. The transcriptional activator p53 is a potent tumor suppressor and about half of all tumors express mutant forms of p53 (Kandoth *et al.* 2013). Five of the seven most common tumor-derived missense p53 mutations affect Arg residues [“IARC p53 Database (p53.iarc.fr)”]. Accordingly, Arg-mutant forms of p53 have been studied extensively, including numerous attempts to rescue function using small molecules that occupy voids created by Arg substitutions (reviewed in Loh 2020). We were particularly interested in p53(R249S) due to the identification (Brachmann *et al.* 1998b) of a second-site suppressor mutation, H168R, in which the “missing” Gdm group of Arg249 is structurally replaced by the “new” Gdm group of Arg168 (Joerger *et al.* 2005) (Figure 4A). The ability of a Gdm group to occupy a void “*in trans*” despite the presence of a Ser side chain was particularly intriguing, since most previous examples of chemical rescue by GdnHCl involved Arg substitutions to Ala or Gly. We hypothesized that free Gdm might bind in the void created by the R249S substitution to restore conformational stability to the DNA-binding domain and rescue p53(R249S) function *in vivo*. Using a yeast *URA3* assay for p53 function equivalent to the one used to identify H168R as a suppressor (Figure 4B), we confirmed that p53(R249S) acts as a null allele (Figure 4C). However, addition of 3 mM GdnHCl to the medium had no discernable effect (Figure 4C).

We suspected that the His168 side chain might interfere with occupancy of Gdm in the R249S void, but GdnHCl had no effect on the function of p53(H168A R249S) (Figure 4C). Finally, we considered that H168 was isolated as a second-site suppressor of R249S only in the context of a third mutation, T123A, which makes critical but poorly understood contributions to cellular function of the triple-mutant protein (Joerger *et al.* 2005). However, GdnHCl conferred no discernable rescue of activity to p53(T123A H168A R249S) (Figure 4C). We suspect that in the case of p53(R249S) and derivatives thereof, the chemical environment surrounding the former site of Arg249 is not conducive to occupancy by free Gdm to an extent that allows the DNA-binding domain to achieve an active conformation long/often enough to confer function, at least to detection limits of our assays.

### Unbiased chemical genetic screens identify other cases of *in vivo* rescue by GdnHCl

**Figure 5:**
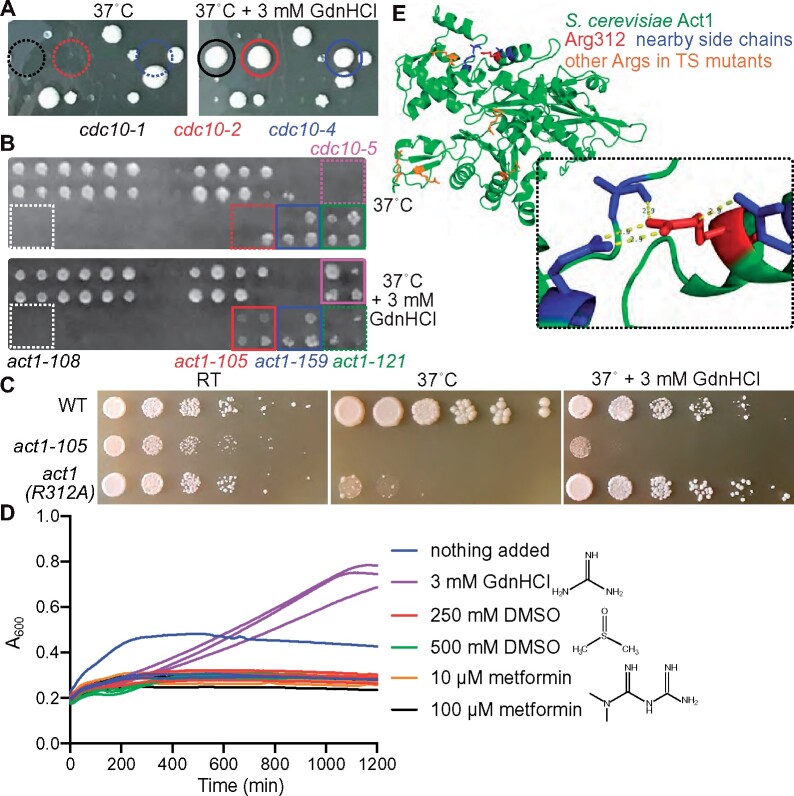
Chemical rescue by GdnHCl of Arg-mutant actin. (A) Cells from a collection of TS mutants were pinned using a screening robot to rich (YPD) plates with or without 3 mM GdnHCl and incubated at 37˚ prior to imaging. Circles are color-coded with text below to indicate specific mutants. Dashed circles indicate failure to form a colony. (B) As in (A) but imaging a portion of a different plate, and using squares to indicate specific mutants. (C) Dilution series of WT (BY4741) or *act1-105* cells on rich (YPD) medium at the indicated temperatures (“RT,” room temperature) with or without GdnHCl for 3 days. (D) 100-µl cultures of *act1-105* cells in rich (YPD) medium with the indicated additives (three cultures for each condition) were incubated with constant shaking at 37˚ in a 96-well plate and the absorbance at 600 nm (“A_600_”) was measured every 5 minutes. (E) Crystal structure of yeast Act1 (PDB 1YAG) showing the location of Arg 312 and nearby residues, and other Arg residues substituted in other TS mutants present in the collection we screened. Inset shows the distances (all 2.9 Å) between atoms in the guanidino group and nearby amino acids, representing presumed intramolecular contacts.

To identify other examples of GdnHCl rescue of mutant proteins using an unbiased approach, we screened two collections of TS mutants for high-temperature colony growth in the presence and absence of 3 mM GdnHCl. One collection of 684 strains included four *cdc10* mutants, which served as convenient positive controls (Figure 5A). It total, 11 of 684 mutants (1.6%) showed detectable evidence of rescue. The *cdc10* mutants showed the greatest degree of rescue; the next most obvious was *act1-105* (Figure 5, A and B). The *act1-105* allele encodes a mutant of the sole yeast actin with two adjacent charged residues, Glu311 and Arg312, substituted to Ala (Wertman *et al.* 1992). The double-mutant cells proliferated slightly slower than WT on rich medium at room temperature (RT; ∼22˚, see Supplementary Figure S3) and not at all at 37˚ unless GdnHCl was added (Figure 5C). Rescue of *act1-105* proliferation at 37˚ by GdnHCl was also observed in liquid culture and was specific to GdnHCl, as we saw no effect of metformin or DMSO, two small molecules with similar chemical composition (Figure 5D). The equivalent Arg residue in human cardiac actin, Arg312, is frequently mutated in dilated cardiomyopathy (DCM), a hereditary cardiac condition (Reza and Owens 2020). *act1(R312H)* mutants are TS (Wong *et al.* 2001). Hence, we focused on the R312A mutation and found that it alone had no effect on proliferation at RT but caused a strong TS defect that was completely rescued by 3 mM GdnHCl (Figure 5C). Arg312 makes multiple intramolecular contacts in yeast Act1 (Figure 5E). When synthesized *in vitro*, human cardiac actin with the R312H substitution displays prolonged interactions with chaperone proteins essential for actin folding (Vang *et al.* 2005). These data are thus consistent with a model in which the Gdm ion binds at the site of the “missing” Arg at position 312 and restores intramolecular contacts that allow the mutant protein to fold, escape chaperone sequestration, and function at high temperature.

**Figure 6:**
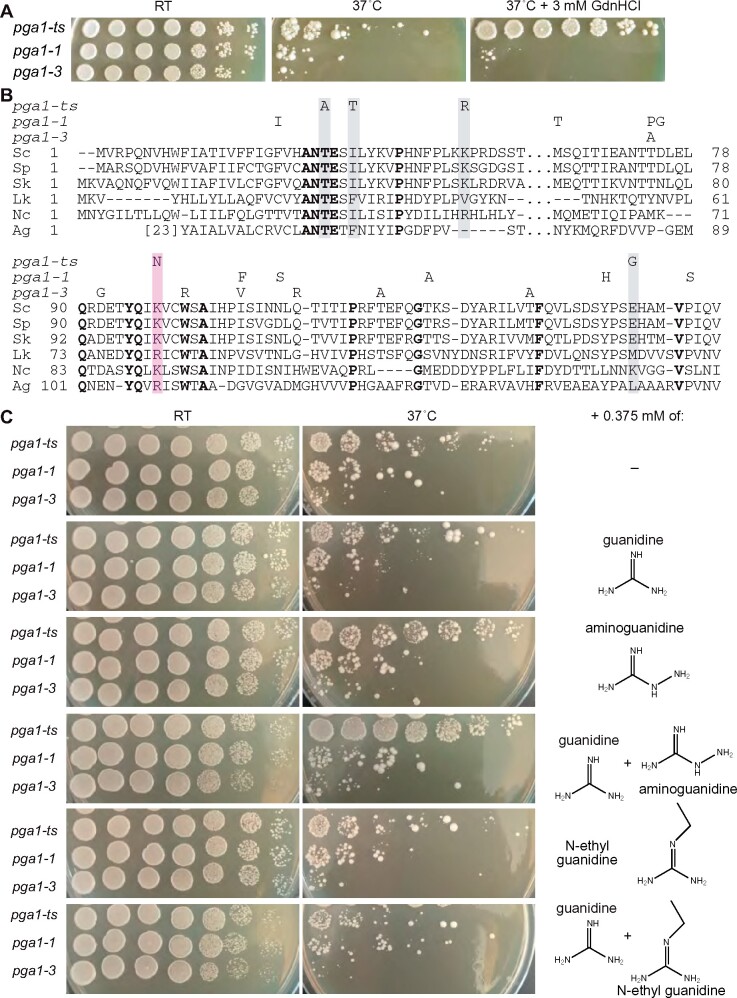
GdnHCl rescue of a Lys-mutant allele of Pga1. (A) Dilution series on rich (YPD) medium with or without 3 mM GdnHCl at the indicated temperatures for strains carrying the indicated alleles of *PGA1*. Strains were H06474, KSY182, and AKY19. (B) Sequence alignment of Pga1 sequences from various fungal species, obtained from the Yeast Genome Database and NCBI server and aligned using COBALT. Residues identical in all WT sequences are in bold. Substitutions in TS mutant ScPga1 alleles are indicated above, those in Pga1-ts are highlighted by shading. Pink, K98N substitution in Pga1-ts at a position where Arg is found in other species. Sc, *S. cerevisiae*; Sp, *S. paradoxus*; Sk, *S. kudriavzevii*; Lk, *Lachancea kluyveri*; Nc, *Naumovozyma castelli*; Ag, *Ashbya gossypii*. (C) As in (A) but with, where indicated, 0.375 mM of GdnHCl (“guanidine”) or derivatives thereof. Where multiple drugs were added, the concentration of each was 0.375 mM.

In another TS collection, we screened 531 mutants and found a single, very clear example of GdnHCl rescue (0.18% of the mutants screened), for an allele of the *PGA1* gene (hereafter “*pga1-ts*”) (Figure 6A). Pga1 encodes a fungi-specific subunit of the essential GPI-mannosyltransferase II (Sato *et al.* 2007). We sequenced the *pga1-ts* allele and found five mutations predicted to result in amino acid substitutions: T25A, I28T, K40R, K98N, and E147G (Figure 6B). To test if GdnHCl suppression was specific to the *pga1-ts* allele, we obtained two additional TS *pga1* alleles, *pga1-1* and *pga1-3*, each of which carries multiple distinct amino acid substitutions (Figure 6B) (Sato *et al.* 2007). GdnHCl at 3 mM failed to suppress the TS colony growth defect of *pga1-1* or *pga1-3* cells (Figure 6A). The GdnHCl derivatives aminoguanidine hydrochloride (“aGdnHCl”) and N-ethylguanidine hydrochloride (“eGdnHCl”) provide partial rescue of other Arg-mutant proteins *in vitro* in a manner that relates to the size of each molecule and thus presumably to steric compatibility with the site of binding (Rynkiewicz and Seaton 1996). We previously showed that, relative to guanidine, aGdnHCl and eGdnHCl are less able to rescue the *cdc10* TS phenotype (Johnson *et al.* 2020). At 0.375 mM, aGdnHCl provided a barely detectable level of rescue of *pga1-ts* growth at 37˚, while GdnHCl and eGdnHCl had no discernable effect (Figure 6C). However, a cocktail of GdnHCl and aGdnHCl at a total concentration of 0.75 mM supported nearly WT colony growth by *pga1-ts* cells (Figure 6C). The GdnHCl/eGdnHCl cocktail had no such effect (Figure 6C). The *pga1-1* and *pga1-3* strains showed no response to any single drug or cocktail (Figure 6C). These results are consistent with a model in which Gdm and, to a greater extent, aGdm make specific molecular contacts with the Pga1-ts protein that restore high-temperature function.

**Figure 7:**
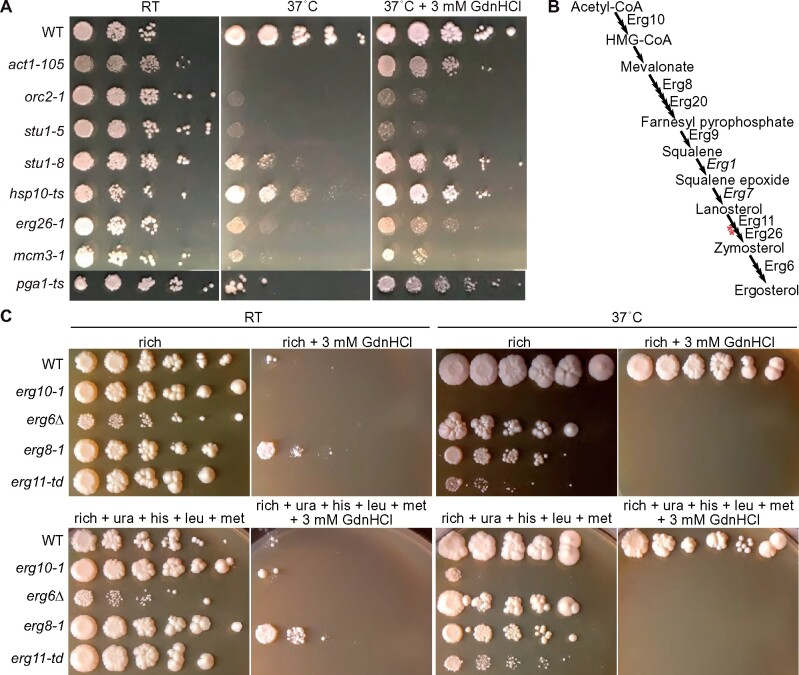
Rescue of *erg26* mutant by GdnHCl and sensitivity of WT cells and other *erg* mutants points to GdnHCl inhibition of ergosterol biosynthesis. (A) TS mutants rescued by GdnHCl identified by screening large collections. Dilution series of cells of the indicated genotypes on rich medium (YPD) with or without GdnHCl at the indicated temperatures (“RT,” room temperature). The *pga1-ts* cells were grown on a different part of the same plate as the others; the image was cropped and moved to preserve space. Strains were: “WT,” BY4741; “*act1-105*,” CBY07857; “*orc2-1*,” CBY08224; “*stu1-5*,” CBY08183; “*stu1-8*,” CBY09448; “*hsp10-ts*,” CBY10708; “*erg26-1*,” CBY11254; “*mcm3-1*,” RSY723; “*pga1-ts*,” H06474. RSY723 is the original *mcm3-1* strain in which this mutant allele was first studied (Lei *et al.* 2002). (B) Schematic of the budding yeast ergosterol biosynthesis pathway with single reactions indicated as arrows, and labels for enzymes represented by mutants whose response to GdnHCl we tested, or, in italics, mutants known to suppress the TS defect of *erg26-1* mutants. Red asterisks indicate steps at which defects generate toxic intermediates. (C) As in (A) but with the strains of the indicated genotypes and, where indicated, rich medium was supplemented with additional nutrients for which these strains are auxotrophic (uracil, histidine, leucine, and methionine). Strains were: “WT,” BY4741; “*erg10-1*,” CBY09883; “*erg6Δ*,” H06534; “*erg8-1*,” CBY08334; “*erg11-td*,” CBY04662.

Three mM GdnHCl partially rescued the TS phenotype of the *erg26-1* mutant (Figure 7A). The TS phenotype of this mutant results from accumulation of toxic 4-carboxysterol intermediates (Figure 7B) (Baudry *et al.* 2001) and mutations in enzymes acting upstream of Erg26 in the ergosterol biosynthesis pathway decrease levels of the toxic intermediates and suppress the *erg26-1* TS phenotype (Germann *et al.* 2005). Thus, rather than directly ameliorating structural or functional defects of the mutant Erg26 enzyme, GdnHCl might rescue high-temperature proliferation of *erg26-1* cells by inhibiting Erg1, Erg7, and/or other upstream enzymes. If so, mutants in those enzymes should be sensitive to GdnHCl. Indeed, we found GdnHCl sensitivity in *erg8-1*, *erg9-ts*, *erg11-td*, and *erg20-ts* mutants (Figure 7C and see Supplementary Figure S4A; neither *erg1* nor *erg7* was represented in these mutant collections). Ergosterol pathway mutants are more permeable to a variety of molecules (Emter *et al.* 2002; Mukhopadhyay *et al.* 2002). Thus some *erg* mutants might appear GdnHCl sensitive merely because they accumulate higher intracellular concentrations. However, an *erg6Δ* mutation did not reduce the concentration of exogenous GdnHCl required for rescue of the TS phenotype of *cdc10(D182N)* cells (Supplementary Figure S4B). We thus interpret our findings as evidence that GdnHCl inhibits one or more WT ergosterol biosynthesis enzymes acting upstream of Erg26.

GdnHCl toxicity was exacerbated by low temperatures. At our “permissive” temperature, 3 mM GdnHCl inhibited colony growth of WT cells (Figure 7C), whereas at 30˚ the same concentration of GdnHCl had almost no effect (Figure 2A). This effect was most pronounced when cells were diluted serially and spotted onto plates, as opposed to streaked onto plates with a toothpick (Supplementary Figure S4). A cold-sensitive colony growth phenotype was previously observed (at 22˚) for *erg6* mutants and was attributed to a cold-sensitive defect in tryptophan uptake (Gaber *et al.* 1989). We also noticed a cold-sensitive colony growth defect in *erg6Δ* mutants, in that colony growth was slower than WT at RT (Figure 7C) but not at 30˚ (data not shown). Interestingly, the *erg8* strain grew slightly better than WT at RT on 3 mM GdnHCl (Figure 7C), providing further support for a role for membrane sterols in the inhibitory effects of GdnHCl at low temperatures. However, supplementing the rich medium with all nutrients for which our strains are auxotrophic did not improve growth of any strain at RT in the presence of 3 mM GdnHCl (Figure 7C). A fully prototrophic strain was also unable to form colonies on rich medium with 3 mM GdnHCl at RT (Supplementary Figure S4C). Thus inhibition of sterol biosynthesis, but not defective nutrient uptake, may contribute to GdnHCl toxicity in WT cells, particularly at low temperatures.

**Figure 8:**
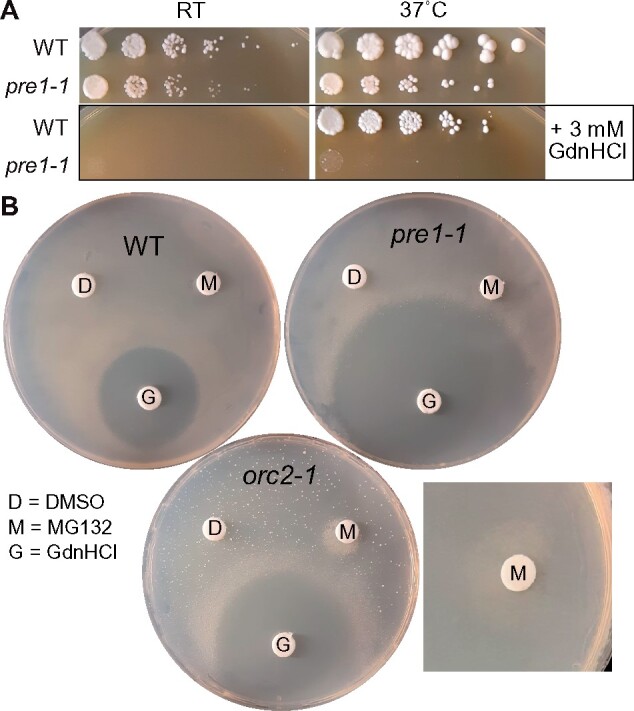
GdnHCl induces proteostatic stress and synergizes with proteasomal inhibition to rescue *orc2-1*. (A) Dilution series of BY4741 (“WT”) and CBY08187 (“*pre1-1*”) plated on rich (YPD) medium with or without 3 mM GdnHCl and incubated at room temperature or 37˚. (B) Cells from liquid rich (YPD) cultures of the indicated strains (“WT,” BY4741; “*pre1-1*,” CBY08187; “*orc2-1*,” CBY08224) were plated to medium containing Pro as the nitrogen source and 0.003% SDS to allow uptake of MG132 (Liu *et al.* 2007). Immediately after plating, paper discs were placed on the plate surface and 10 µl of the indicated chemicals were spotted onto the discs: 100% DMSO, 40 mM MG132 in DMSO, or 5M GdnHCl in water. Plates were then incubated at 37˚ prior to imaging. The square image at right is a close-up of the MG132 disc from an independent experiment with no GdnHCl-soaked disc on the plate, ruling out any contribution from GdnHCl to the observed rescue. The sprinkling of *orc2-1* colonies dispersed across the entire plate reflects non-TS derivatives that arise spontaneously at 37˚ independently of added chemicals and which we have not characterized further. (C) Induction of the Hsf1-dependent cytosolic stress response measured using a strain (YJW1741) expressing RFP (mKate) from the constitutive *TEF2* promoter and GFP from an artificial Hsf1-inducible promoter containing four heat shock elements (4xHSE). Cells (23 per sample) were imaged following overnight growth at RT with or without 3 mM GdnHCl. Error bars, mean ± SEM. “A.U.,” arbitrary units.

Several cases of GdnHCl rescue or sensitivity suggested possible GdnHCl inhibition of proteasome function. First, we found weak GdnHCl rescue of the TS growth defects of *orc2-1* and *stu1-5* mutants (Figure 7A). Proteasome mutations suppress the TS phenotype and ∼10-fold decrease in protein levels caused by the P603L mutation in *orc2-1* (Shimada *et al.* 2002; Costanzo *et al.* 2016). Similarly, no Arg residue is mutated in the *stu1-5* allele (Table 1) but mutant protein levels are reduced (Brew and Huffaker 2002) and various proteasomal mutations provide partial suppression (Costanzo *et al.* 2016). Second, we found that two TS alleles of proteasome subunits—*pre4-ts* (Hilt *et al.* 1993) and *pre1-1* (Heinemeyer *et al.* 1993)—are sensitive to GdnHCl (Figure 8, A and B and Supplementary Figure S4A; note that the restrictive temperature for *pre1-1* is 38.5˚). Pharmacological proteasome inhibition in *S. cerevisiae* has been previously shown to rescue the function of mutant proteins with reduced steady-state levels relative to WT (Singh *et al.* 2010). To test if proteasome inhibition is sufficient to explain the GdnHCl rescue we observed, we used the proteasome inhibitor MG132 with the *orc2-1* mutant in the paper disc assay. In addition to the expected halo of *orc2-1* rescue observed around the GdnHCl-soaked disc, we saw a zone of colony growth next to the MG132-soaked disc (Figure 8B). Thus both GdnHCl and MG132 rescue *orc2-1*. Finally, a very recent study found evidence that the proteasome inhibitor Bortezomib improves proliferation by many of the same TS mutants we screened, including *orc2-1* (Costanzo *et al.* 2021). However, *orc2-1* was the only mutant that responded positively to both GdnHCl and Bortezomib (Costanzo *et al.* 2021). Thus rather than directly inhibiting the proteasome, these findings are most consistent with GdnHCl directly stabilizing conformations of some mutant proteins in ways that allow them to escape proteolytic degradation.

**Table 1:** Summary of mutants rescued by small molecules

Gene	GdnHCl (3 mM)	**TMAO** *a* **(0.5 M)**	DMSO (5%)	Notes, known mutations
*ACT1*	*act1-105*	*act1-111* *act1-129*		*act1-105* (E311A, R312A) *act1-111* (D222A, E224A, E226A) *act1-129* (R177A, D179A)
*ARG3*	*arg3(R69G)*			*arg3(R69G)* not tested for TMAO, DMSO rescue
*ARP2*			*arp2-14*	
*ARP3*		*arp3-D11A* *arp3-G15C* *arp3-H161A*	*arp3-G15C*	
*CDC10*	*cdc10-1* *cdc10-2* *cdc10-4* *cdc10-5*	*cdc10-2*		GdnHCl binds Cdc3, bypassing mutant Cdc10 during septin hetero-oligomerization
*CDC23*			*cdc23-4*	T94M
*ERG26*	*erg26-1*	*erg26-1*		
*HSP10*	*hsp10-ts*			P36H
*KAR2*		*kar2-159*	*kar2-159*	G417S, variable DMSO rescue
*LCB1*		*lcb1-2* *lcb1-4* *lcb1-5*	*lcb1-2* *lcb1-5*	*lcb1-2* (M261R) *lcb1-4* (D320Y) *lcb1-5* (D299Y)
*MCM3*	*mcm3-1*	*mcm3-1*		G246E
*ORC2*	*orc2-1*	*orc2-1* *orc2-2* *orc2-4*		*orc2-1* (P603L)
*PGA1*	*pga1-ts*			T25A, I28T, K40R, K98N, E147G. Not tested for TMAO, DMSO rescue.
*SMC3*		*smc3-1*	*smc3-1*	
*STU1*	*stu1-5* *stu1-8*		*stu1-5* *stu1-6*	*stu1-5* (S88T Q257K T492A F483L Y534H L561S V575A T579S N596I P616L S621T) *stu1-6* (mutation in N-region) *stu1-8* (mutation in mid-region)
*TUB4*		*tub4-ΔDSY*	*tub4-ΔDSY*	Variable DMSO rescue
*URA3*	*ura3(R235A)*			*ura3(R235A)* not tested for TMAO, DMSO rescue

a For a complete list of TMAO rescues, see Supplementary Table S2.

### Chemical rescue in living cells by other naturally occurring small molecules

A model in which guanidine restores native conformations to a variety of mutant proteins, including those without Arg substitutions, is reminiscent of the nonspecific conformational stabilization *in vitro* that has been reported at subdenaturing concentrations for both GdnHCl and urea (Bhuyan 2002). If GdnHCl rescues TS mutant function by conferring general thermal stability, then urea might do the same. However, when we used 10 mM urea to screen a collection of TS mutants we had previously screened for GdnHCl rescue, no mutant showed clear evidence of rescue. Supplementary Figure S5A shows tests of several candidate mutants, each of which ultimately failed to display anything more than the slightest evidence of urea rescue. To address the possibility that 10 mM exogenous urea does not generate enough available intracellular urea, we used the paper disc assay and *orc2-1* cells to test a range of concentrations and also used medium with 500 mM urea to test nine mutants that were rescued by 3 mM GdnHCl. In no case did we find any evidence of urea rescue (Supplementary Figure S6). Thus our findings do not support a model in which GdnHCl provides in living cells the same kind of general conformational stabilization of mutant proteins previously observed *in vitro* with urea.

**Figure 9:**
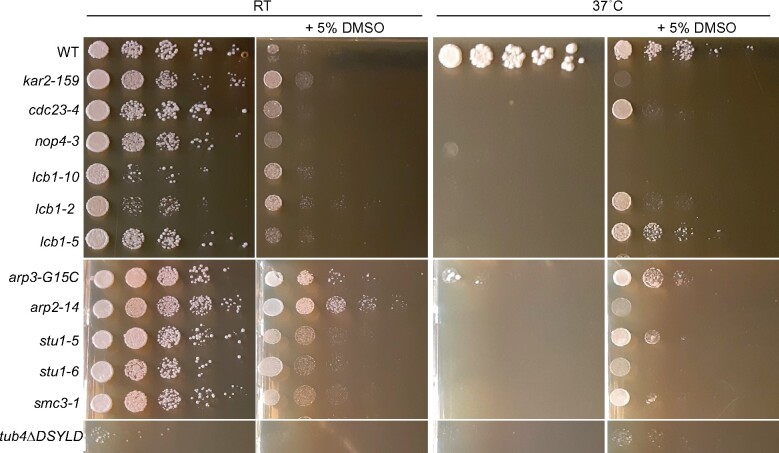
Chemical rescue of TS mutants by DMSO. Dilution series of cells of the indicated genotypes on rich medium (YPD) with or without 5% DMSO incubated at the indicated temperatures (“RT,” room temperature). Strains were: “WT,” BY4741; “*kar2-159*,” CBY07833; “*cdc23-4*,” CBY06436; “*nop4-3*,” CBY10671; “*lcb1-10*,” CBY10175; “*lcb1-2*,” CBY10161; “*lcb1-5*,” CBY10156; “*arp3-G15C*,” CBY08235; “*arp2-14*,” CBY04958; “*stu1-5*,” CBY08183; “*stu1-6*,” CBY10325; “*smc3-1*,” CBY08012; “*tub4ΔDSYLD*,” CBY11146.

We next investigated other naturally occurring small molecules known to alter protein conformations. DMSO is found in many natural sources including fruit (Pearson *et al.* 1981) and marine algae (Lee and Mora 1999). In addition to its laboratory use as a solvent and cryoprotectant due to effects on cellular membranes (Notman *et al.* 2006; Gurtovenko and Anwar 2007; Gironi *et al.* 2020), DMSO also affects protein folding and stability *in vitro* (Rammler and Zaffaroni 1967). Like GdnHCl, DMSO (at 2.5% v/v) cures yeast cells of at least one prion (Tuite *et al.* 1981). While screening various compounds for rescue of a mutant human enzyme in yeast, a previous study discovered that DMSO, intended as a solvent control, itself showed an effect (Singh *et al.* 2007). At 614 mM, mutant function was nearly equal to WT (Singh *et al.* 2007). We first tested for rescue of the TS phenotype of *act1(E311A R312A)* by DMSO at 250 and 500 mM and saw no effect (Figure 5E). We next screened a collection of 684 TS mutants for rescue by 705 mM (5% v/v) DMSO. Ten mutants (1.5% of the mutants screened) representing nine genes formed colonies at 37˚ only in the presence of 5% DMSO (Figure 9A). The *kar2-159* allele (substitution G417S) affects the yeast homolog of mammalian BiP (Binding immunoglobulin Protein), an Hsp70-family ER chaperone (Vogel *et al.* 1990). To ask if DMSO generally bypasses the requirement for Hsp70 chaperones in the folding of essential proteins, we tested two strains, one in which three of the four *SSA* (Stress-Seventy subfamily A) cytosolic Hsp70 genes are deleted and only WT *SSA1* remains, and an otherwise isogenic strain carrying a TS allele of *SSA1* (Becker *et al.* 1996). 5% DMSO failed to rescue the *ssa1-ts* strain and blocked colony growth by the *SSA1* strain at 37˚ (Supplementary Figure S7). These results are consistent with a model in which DMSO alters protein conformations, but in ways that do not generally substitute for the action of Hsp70 chaperones.

Several mutants rescued by DMSO represent proteins directly or indirectly involved in lipid synthesis and membrane dynamics. Lcb1 (*lcb1-2* and *lcb1-5*, Figure 9A) is a serine palmitoyltransferase required for sphingolipid synthesis (Buede *et al.* 1991). The substitutions in these alleles are distinct (Table 1) and other *lcb1* mutants in the collection were not rescued (Figure 9A and data not shown), suggesting that DMSO does not specifically act on one mutant form of the protein and also that DMSO does not prevent or counteract the effects of Lcb1 dysfunction in general. Arp2 and Arp3 are 30% identical, actin-related proteins that are essential subunits of a heptameric complex (Welch *et al.* 1997). While *arp2-14* is the only allele of *ARP2* in the collection we screened, *arp3-G15C* was the only of six *arp3* alleles to be scored as rescued by DMSO in our screen (Table 1, Supplementary Table S1). It is unclear why DMSO rescue was specific to *arp3-G15C*, though the study that characterized these *arp3* mutants found a unique combination of phenotypes for *arp3-G15C* with regard to sensitivity to temperature, 0.9 M NaCl, and 3% formamide (Martin *et al.* 2005). The final category of TS mutants rescued by DMSO—*stu1-5, stu1-6* (Yin *et al.* 2002)*, tub4ΔDSYLD* (Vogel and Snyder 2000), *cdc23-4* (Melloy and Holloway 2004), and *smc3-1* (Tanaka *et al.* 2000)—are linked to microtubule function, which in yeast is mostly restricted to the mitotic spindle. We suspect that rescue of these mutants is an indirect effect of DMSO effects on microtubules (see Discussion).

**Figure 10:**
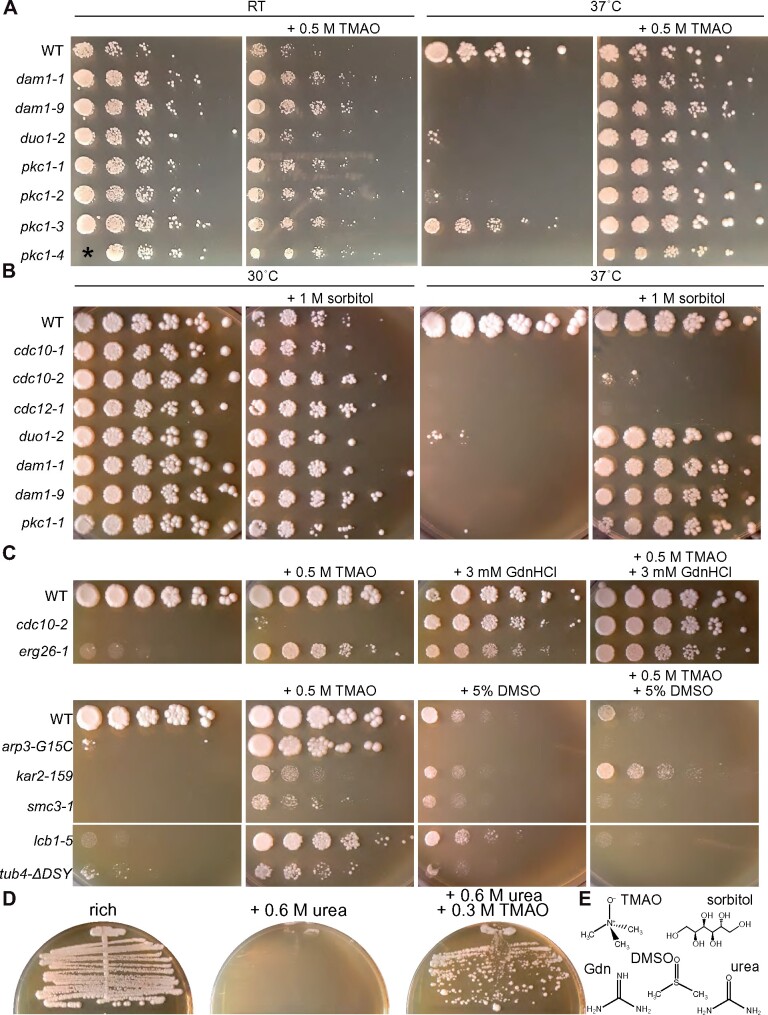
Chemical rescue of TS mutants by TMAO. (A) Dilution series of cells of the indicated genotypes on rich medium (YPD) with or without 0.5 M TMAO incubated at the indicated temperatures (“RT,” room temperature). Strains were: “WT,” BY4741; “*dam1-1*,” CBY07974; “*dam1-9*,” CBY07966; “*duo1-2*,” CBY07958; “*pkc1-1*,” CBY09957; “*pkc1-2*,” CBY09962; “*pck1-3*,” CBY09972; “*pkc1-4*,” CBY09967. The asterisk indicates where, due to a technical error, the undiluted *pkc1-4* sample was not applied to the plate. (B) As in (A) but with different mutants and 1 M sorbitol instead of TMAO. Strains were: “WT,” BY4741; “*cdc10-1*,” CBY06417; “*cdc10-2*,” CBY06420; “*cdc12-1*,” CBY05110; “*duo1-2*,” CBY07958; “*dam1-1*,” CBY07974; “*dam1-9*,” CBY07966; “*pkc1-1*,” CBY09957. (C) As in (A) but with different mutants, 0.5 M TMAO, 3 mM GdnHCl and/or 5% DMSO, and all plates were incubated at 37˚. Strains were: “WT,” BY4741; “*cdc10-2*,” CBY06420; “*arp3-G15C*,” CBY08235; “*kar2-159*,” CBY07833; “*smc3-1*,” CBY08012; “*lcb1-5*,” CBY10156; “*tub4ΔDSYLD*,” CBY11146. (D) BY4741 was streaked on rich (YPD) medium with or without 600 mM urea or 300 mM TMAO and incubated at 37˚ for 4 days prior to imaging. (E) Chemical structures of small molecules were used in this study.

Studies with mutant alleles of human cystathionine β-synthase expressed in yeast found that some mutant proteins could be rescued by DMSO or TMAO, whereas other mutants could be rescued only by TMAO (Singh *et al.* 2007). We screened the same collection of 684 TS mutants with 0.5 M TMAO at 37˚ and found 131 candidate examples of rescue (19.2% of the mutants screened), six of which were also rescued by DMSO (Table 1, Supplementary Table S2 and Figures S7C and S8). Put another way, DMSO rescued <5% of the mutants rescued by TMAO, but TMAO rescued over half of the mutants that were rescued by DMSO. We also found 25 candidate examples that showed some degree of sensitivity (3.7% of the mutants screened; Supplementary Table S2). Validating all the candidates by dilution series would have been impractical, so we tested only a subset (Figure 10 and Supplementary Figure S8A). Four alleles of *PKC1*, which encodes a kinase crucial for cell wall integrity, are known to be rescued by a variety of osmolytes (Levin and Bartlett-Heubusch 1992; Huang and Symington 1994); higher osmolarity of the medium counteracts intracellular turgor pressure and prevents cell lysis when cell walls are structurally compromised. Each of these *pck1* mutants was also rescued by TMAO (Figure 10A). One allele of *cdc10* showed weak rescue by TMAO (Table 1 and see Figure 10C). Septins are also required for proper cell wall biogenesis (DeMarini *et al.* 1997; Cid *et al.* 2001), and two other septin-mutant strains, *cdc12-1* and *cdc12-6*, are also known to be partially rescued by the osmolyte sorbitol (Merlini *et al.* 2015). Because all TS septin mutants show similar cellular defects at the restrictive temperature (Hartwell 1971), we suspected that TMAO rescue of *cdc10-2* may also represent a case of “osmotic support.” Indeed, at 37˚ 1 M sorbitol rescued *pkc1-1* and *cdc10-2* (Figure 10B). However, it was unclear why the *cdc12-1* mutant and other *cdc10* mutants in the collection we screened did not show TMAO rescue. Sorbitol rescue of *cdc12-1* in the published study was only apparent up to 34˚ (Merlini *et al.* 2015), and we confirmed that at 37˚ 1 M sorbitol failed to rescue the *cdc12-1* mutant present in the collection (Figure 10B). We assessed a range of temperatures in liquid cultures and found that *cdc10-2* has a higher permissive temperature than *cdc12-1* and the other *cdc10* mutants in the collection (Supplementary Figure S8B). Thus only for *cdc10-2* did moderate TMAO rescue exceed the threshold of detection in our screen. Like TMAO (Figure 10A), 1 M sorbitol also rescued *dam1-1*, *dam1-9*, and *duo1-2* (Figure 10B). Of 97 mutants that we found to be partially rescued by TMAO and were also analyzed in the very recent Costanzo *et al.* (2021) study, 29 (30%) showed evidence of rescue by sorbitol. By contrast, only 12% (533 of 4429, two-sided Fisher’s exact test *P*-value < 0.0001) of all the mutants screened by Costanzo *et al.* showed sorbitol rescue. Thus TMAO and sorbitol likely share a common mechanism in their ability to rescue many TS mutants.

Studies of mutant human protein function in yeast found that a cocktail including TMAO, DMSO, and sorbitol appeared to have a synergistic rescue effect (Singh *et al.* 2007). We tested cocktails of TMAO plus GdnHCl and TMAO plus DMSO on selected mutants and found that in some cases the combinations provided approximately additive positive effects on mutant proliferation (*e.g.*, TMAO plus GdnHCl for *cdc10-2*) whereas in others (*e.g.*, TMAO plus DMSO for *lcb1-5*) the combination eliminated rescue altogether (Figure 10C). Finally, given the apparent effectiveness of TMAO in rescuing TS mutant protein function, and the natural accumulation of TMAO in organisms with high intracellular urea, we predicted that if the toxic effects of high concentrations of exogenous urea on *S. cerevisiae* reflect misfolding or denaturation of essential proteins, then TMAO might counteract these effects and restore viability. To test this prediction, we streaked WT haploid cells on rich medium with 600 mM urea in the absence or presence of 300 mM TMAO, a concentration chosen to mimic the 2:1 urea: TMAO ratio found in many marine organisms (Yancey and Somero 1979). Urea completely blocked colony formation at 37˚ and the addition of TMAO almost completely reversed this effect (Figure 10D). While other explanations are possible, this result is consistent with a model in which exogenous urea denatures proteins and TMAO counteracts this effect, as occurs in the organisms inside which both molecules are found naturally.

## Discussion

### Why does GdnHCl rescue only some Arg-mutant proteins *in vivo*?

Our unbiased screens of large collections of mutants and our targeted tests of Arg-mutant proteins illustrate just how lucky we were to identify GdnHCl rescue of *cdc10* mutants (Johnson *et al.* 2020). Only two other mutants, *pga1-ts* and *act1(R312A)*, showed a similar extent of GdnHCl rescue. None of ∼30 other Arg-to-small-side-chain mutants in the same TS collection (Supplementary Table S1) or 19 other *ACT1* mutants in the collections we screened, including six with Arg-to-Ala substitutions (Figure 5D, Supplementary Table S1), showed any evidence of GdnHCl rescue (Figure 5B and data not shown). Do our successes and failures provide any mechanistic insights?

Some Arg residues required for protein function *in vitro* are dispensable *in vivo*, as we found for β-gal(R599A). Arg at this position is missing in multiple β-galactosidases with known *in vivo* X-gal activity (Supplementary Figure S1E) (Hall 1976; Schmidt *et al.* 1989) (Supplementary Figure S2C). Hence, despite the apparent importance of this residue for β-gal activity on *o*NPG *in vitro*, it is not very important *in vivo*. For Arg mutation in enzymes that did cause *in vivo* phenotypes, amenability to rescue may reflect the extent to which the Arg mutation weakens the affinity of the enzyme for the relevant substrate. K_M_, the substrate concentration at which a reaction rate is half-maximum, is a reliable indicator of this affinity. For the kinase Src, the R388A substitution increased the K_M_ for ATP only about two-fold (81–182 µM), allowing *in vivo* rescue by a nontoxic concentration of imidazole (Qiao *et al.* 2006). The R57G mutation in EcOTC increases the K_M_ by about five-fold (50–260 µM), whereas the R235A mutation in Ura3 increases the K_M_ of the ODCase reaction over 1000-fold (1.4–1800 µM). These differences correlate with the extent of *in vivo* GdnHCl rescue. Furthermore, the high entropic cost of properly ordering an additional ligand, Gdm, in the active site of a mutant enzyme likely constrains the extent of chemical rescue to a fraction of the activity of a WT enzyme, where the functional group(s) are tethered to the polypeptide backbone in the context of a side chain. Indeed, the *in vitro* study estimated that restoring Ura3(R235A) activity to that of WT would require an effective Gdm concentration of 160 M (Barnett *et al.* 2010). Our findings provide evidence that for mutant enzymes in which the mutation affects substrate binding, chemical rescue by GdnHCl *in vivo* is possible only when two conditions are met: (1) the mutant enzyme has a sufficiently low substrate affinity in the absence of Gdm such that, at the available intracellular substrate concentration, activity is perturbed enough to generate a detectable phenotype, and (2) Gdm at nontoxic intracellular concentrations binds well enough to increase the affinity to a point that sufficient substrate can be converted to product to generate a rescue phenotype. For nonenzyme proteins like Act1(R312A), Gdm binding comes at an entropic cost but other contacts are intramolecular, hence full rescue is more achievable.

From this perspective, yeast Asn1(R344A) was a good candidate. Asparagine synthetase B has multiple substrates (ATP, glutamine, and aspartate). The R325A mutation in the *E. coli* enzyme slightly decreases the K_M_ for glutamine and increases the K_M_ for ATP just over twofold (Boehlein *et al.* 1997). We found a strong *in vivo* phenotype for Asn1(R344A) but no *in vivo* rescue by GdnHCl. A recent study also examined the Asn1(R344A) mutant in the context of the filaments that Asn1 and Asn2 form in nutrient-deprived cells, the functional significance of which remains unknown (Noree *et al.* 2019). Asn1(R344A) was unable to form filaments (Noree *et al.* 2019), suggesting either that enzymatic activity is required for polymerization or that Arg 344 is required for both polymerization and activity. Structures of *E. coli* (Larsen *et al.* 1999) and human (Zhu *et al.* 2019) asparagine synthetase solved after the *in vitro* GdnHCl rescue study revealed that the Arg 344 equivalent in both enzymes projects away from the substrate-binding sites and toward solvent, at least in the dimeric crystal form (Figure 3A). It is thus unclear how Arg in this position contributes to substrate binding or enzymatic activity, and the inter- or intra-molecular contacts made by the guanidino group remain unknown. That GdnHCl rescues activity of the mutant protein *in vitro* but not *in vivo* may simply reflect a role for this Arg residue that is not recapitulated *in vitro*. Alternatively, since in the *in vitro* rescue study the purified enzyme was synthesized in the absence of GdnHCl, in our *in vivo* study GdnHCl is present during Asn1(R344A) synthesis and may alter its folding environment (kinetics, aggregation propensity, and chaperone expression/activity) in a way that precludes its function.

### Rescue of non-Arg mutants by GdnHCl

Our unbiased screening approach pointed us to mutants for which the mechanism of GdnHCl rescue is less obvious, such as *pga1-ts*. There is no structural information for Pga1 or any homolog, and *ab initio* structure predictions for this transmembrane protein generate low-confidence results (unpublished observations). However, only two of the substitutions in the Pga1-ts mutant protein affect highly conserved residues, and one of these, K98N, substitutes to Asn a Lys residue that is Arg in the Pga1 homologs from *Lachancea kluyveri* and *Ashbya gossypii* (Figure 6B). Lys sidechains are, like those of Arg, long and positively charged at physiological pH. Thus we speculate that Gdm provides molecular contacts that are missing in the Pga1-ts protein due to the K98N substitution, and thereby restores function to the mutant cells.

We note that in the crystal structure of one Arg-mutant human protein (mitochondrial aldehyde dehydrogenase R475Q) a Gdm ion occupies the position of a Gdm group not from the “missing” Arg, but from another Arg that was mispositioned due to the mutation (Larson *et al.* 2007). Thus in principle, GdnHCl could rescue various non-Arg-mutant proteins in which a positively-charged side chain (of Arg, Lys, or even His) is missing from a particular location as a direct or indirect result of a mutation. This mechanism may explain GdnHCl rescue of other mutants with non-Arg substitutions, such as *mcm3-1* (substitution G246E), which is suppressed by overexpression of another subunit of the MCM complex (Yan *et al.* 1991), consistent with a misfolded oligomerization interface. This rescue mechanism may also apply to the P36H substitution in Hsp10 (Figure 7A), a subunit with Hsp60 of the chaperone that refolds numerous proteins following their import into mitochondria (Höhfeld and Hartl 1994). The mutant protein shows normal thermal stability and overexpression does not improve function (Dubaquié *et al.* 1997). Instead, elevated temperature appears to perturb allosteric communication with Hsp60 (Dubaquié *et al.* 1997). Thus Gdm may directly act on Hsp10(P36H) or even Hsp60 to restore allosteric communication. Alternatively, if there is a single essential client of Hsp10–Hsp60 that misfolds in the mutant cells at 37˚, Gdm might bind to it and directly promote acquisition of the native conformation, acting as a true “chemical chaperone.” Finally, since Gdm likely inhibits the mitochondrial homolog of Hsp104, Hsp78, it is possible that in *hsp10(P36H)* cells at 37˚ Hsp78 inappropriately unfolds some essential protein(s), and Hsp78 inhibition thereby restores viability. We find this mechanism unlikely because *hsp78Δ* does not rescue *hsp10(P36H)* (Costanzo *et al.* 2016). We note that *hsp104Δ* displays positive interactions with 10 TS mutants but not any of those we found to be rescued by GdnHCl (Costanzo *et al.* 2016), strongly suggesting that in no case was GdnHCl inhibition of Hsp104 responsible for rescue.

### “Off-target” effects of GdnHCl

Under normal growth conditions, GdnHCl is toxic to WT yeast cells at concentrations ≥5 mM (Tuite *et al.* 1981). Some of the GdnHCl-sensitive mutants that we identified at 3 mM GdnHCl may be explained if Gdm occupies voids created by substitutions and exacerbates mutant protein dysfunction. However, functional connections between GdnHCl-sensitive mutants also point to GdnHCl inhibition of specific cellular processes in WT cells, ergosterol synthesis being the clearest candidate (see Results). We also found that *inn1(F59S K210E)* cells are GdnHCl-sensitive at 37˚ (Supplementary Figure S4A). Another *inn1* TS mutant displays negative genetic interactions with TS alleles of multiple septin genes (Costanzo *et al.* 2016). We suspect that *inn1* sensitivity to GdnHCl reflects subtle GdnHCl-induced defects in the efficiency of septin octamer assembly at 37˚ that have no overt consequence in WT cells. GdnHCl sensitivities in *med6(K39R N43S M69V K75T R173G N252S D281G)*, *mpe1-ts*, *rad3(C590Y)*, and *ipa1-ts* mutants (Supplementary Figure S4A) may point to some general problem with transcription and/or mRNA processing in GdnHCl-treated cells, considering that Med6 is part of the RNA Pol II mediator complex (Lee *et al.* 1997), Mpe1 (Vo *et al.* 2001) is a subunit of the CPF mRNA cleavage and polyadenylation factor, which interacts physically and genetically with Ipa1 (Costanzo *et al.* 2016), and Rad3 is a subunit of RNA Pol II initiation factor TFIIH (Feaver *et al.* 1993).

### Clues about the mechanisms of DMSO rescue from the function of rescued mutants

DMSO resembles Gdm but there was little overlap in the mutants rescued by each molecule, suggesting distinct mechanisms of action. Gene expression analysis of *S. cerevisiae* cells exposed to 1 M DMSO found that Kar2 transcript levels increase slightly (Momose *et al.* 2010). Hence a trivial explanation for *kar2(G417S)* rescue is that elevated levels of the mutant protein compensate for its dysfunction. Addition of DMSO to *S. cerevisiae* is known to upregulate lipid synthesis genes, resulting in increased phospholipid content in membranes, which presumably counteracts direct membrane effects of the drug (Murata *et al.* 2003). Accordingly, DMSO may alter membrane properties to something compatible with proliferation despite the sphingolipid defects in *lcb1* mutants. However, sphingoid bases also act as signaling molecules and thereby influence many cellular processes, including the response to high temperature itself: in cells carrying a distinct TS allele of *LCB1* that was not present in the collection we screened, proliferation at 37˚ is restored by overexpressing ubiquitin, which appears to promote proteasomal clearance of misfolded proteins without restoring normal sphingolipid content (Friant *et al.* 2003). Thus DMSO could rescue *lcb1* mutants in a number of ways among which our current data are unable to distinguish. In cultured mammalian cells, DMSO and other amphiphilic compounds alter membrane tension and thereby increase the rate of endocytosis (Raucher and Sheetz 1999). The Arp2/3 complex nucleates actin filaments and is required for the motility of cortical actin patches and, in turn, the internalization step of endocytosis (Winter *et al.* 1997). Hence DMSO alteration of membrane tension may facilitate endocytic events that are otherwise stalled at the plasma membrane in Arp2/3 mutants. Interestingly, *arp2-14* and *arp3-G15C* cells also proliferated better than WT at RT in the presence of 5% DMSO (Figure 9A). Thus DMSO may synergize with low temperature to alter membrane properties in a way that perturbs endocytosis and is counteracted by subtle changes in actin patch motility in these *arp* mutants. A previous screen of the *S. cerevisiae* homozygous diploid deletion collection identified nine mutants that were more resistant than WT to 8% DMSO (Zhang *et al.* 2013), none of which has any obvious functional relationship to endocytosis or membrane tension. However, a recent study found that *chm7Δ* also confers improved colony growth in the presence of 4% DMSO, which presumably relates to Chm7’s function in membrane dynamics in the ER and nuclear envelope (Capella *et al.* 2020). Why loss of Chm7 would ameliorate effects of DMSO on membrane properties remains unclear.

DMSO exerts profound effects on purified tubulin *in vitro*, lowering the critical concentration for microtubule polymerization (Algaier and Himes 1988) and lifting the requirement for microtubule-associated proteins (Himes *et al.* 1976). In cultured mouse oocytes, exposure to ∼10% DMSO rapidly induced the formation of cold-resistant microtubules (Johnson and Pickering 1987). We speculate that direct DMSO effects on microtubule stability/dynamics underlie chemical rescue of a group of mutants. Stu1 directly binds microtubules and mutations in tubulin subunits suppress the TS defect of *stu1-5* mutants (Yin *et al.* 2002). *TUB4* encodes gamma tubulin, which nucleates microtubules, and drugs that alter the stability of microtubules rescue the TS phenotype of the *tub4ΔDSYLD* mutant (Vogel and Snyder 2000). (We note that DMSO rescue of *tub4ΔDSYLD* was subtle at best and altogether absent in some experiments.) Cdc23 is a subunit of the anaphase-promoting complex that localizes to microtubule ends (Melloy and Holloway 2004). Smc3 is a subunit of the cohesin complex that holds sister chromatids together, contributing to tension that is crucial for stable microtubule–kinetochore attachments (Tanaka *et al.* 2000). In a recent study, Ming Sun *et al.* (2020) identified a genetic interaction between *smc3-1* and *irc15Δ*, which eliminates a microtubule-binding protein that regulates microtubule dynamics (Keyes and Burke 2009). Thus our findings do not support a model in which DMSO binds directly to many mutant proteins, and instead point mainly to indirect rescue mechanisms.

### TMAO: osmolyte, molecular crowding agent, or anti-denaturant?

Among the molecules we examined, TMAO was by far the most effective at rescuing the functions of a range of mutants. Might we have found even more examples of TMAO rescue had we screened at lower temperatures? Comparison of the annotated restrictive temperatures of the mutant collection as a whole with those that showed apparent TMAO rescue at 37˚ suggested a slight bias toward mutants with restrictive temperatures closer to 37˚, but not a strict threshold below which no rescue is possible (Supplementary Figure S7C). Thus while it appears to act quite generally, TMAO does not universally improve mutant protein function. Indeed, some mutants were inhibited by TMAO (see Supplementary Table S2 and Figure S8 and the *brl1-2221* and *brl1-3231* mutants in Supplementary Figure S7A), consistent with TMAO destabilization of other proteins *in vitro* (Singh *et al.* 2005) and *in vivo* (Bandyopadhyay *et al.* 2012).

The overlap in mutants rescued by sorbitol and TMAO despite their very different chemical structures (Figure 10E) suggests that many of these cases represent either osmotic support to prevent lysis of fragile mutant cells or general “molecular crowding” that stabilizes thermolabile proteins. Like DMSO, TMAO directly promotes microtubule polymerization and stabilizes microtubules against depolymerization (Sackett 1997; Chase *et al.* 2017; Bachand *et al.* 2018). Multiple studies in the literature observed rescue of TS mutants with spindle defects by sorbitol (Korolyev *et al.* 2005; Gerson-Gurwitz *et al.* 2009), and sorbitol provides resistance to WT cells against drugs that destabilize microtubules, leading to the idea that molecular crowding stabilizes microtubules (Korolyev *et al.* 2005). This effect likely explains a class of mutants: TMAO rescued *tub4ΔDSLYD* (Figure 10C), *duo1-2* and multiple distinct TS alleles of *DAM1* (Figure 10A, Table 1, Supplementary Table S2). Dam1 and Duo1 are subunits of the Dam1 complex (also called DASH) that links kinetochores to the microtubules of the mitotic spindle (Hofmann *et al.* 1998; Westermann *et al.* 2005). Considering the number and diversity of TMAO-rescued mutants, however, we suspect that some reflect direct stabilizing effects of TMAO on mutant protein conformation.

Combining small molecules had various effects. Using the additive effect of GdnHCl and TMAO on *cdc10-2* as an example (Figure 10C), Gdm binding to another septin protein drives assembly of septin complexes that bypass the mutant Cdc10 proteins, but these complexes are not fully functional, leading at 37˚ to slight cellular defects (Johnson *et al.* 2020) which are likely remedied by the osmotic support TMAO provides. By contrast, TMAO and DMSO neutralized each other in *lcb1-5* cells (Figure 10C). TMAO might change the conformation of the Lcb1(D299Y) protein or its reaction kinetics in a way that increases serine palmitoyltransferase activity, but the resulting lipid composition may be incompatible with the direct membrane effects of DMSO. Finally, the fact that TMAO neutralized the toxic effects of 600 mM urea on WT cells strongly argues that in this concentration range enough intracellular urea is available to denature proteins (and other molecules, Gluick and Yadav 2003). Thus, the failure of 500 mM urea to rescue any mutant we tested further supports our conclusion that GdnHCl and urea have distinct effects *in vivo*, consistent with *in vitro* studies (Lim *et al.* 2009).

### Implications for evolution and human disease

In the context of human disease, even the slightest increase in function might improve the quality of life. If GdnHCl has the potential to restore function to even a fraction of Arg-mutant proteins, then it should be considered as a possible therapeutic. Our findings also provide additional insights into likely side effects (*e.g.*, ergosterol/cholesterol synthesis) and conditions in which these effects are most pronounced (low temperature). DMSO and TMAO likely have their own direct effects on specific cellular processes TMAO also appears to act in general ways that ameliorate functional defects in multiple mutant proteins. DMSO is found in many natural foods (Pearson *et al.* 1981) and has a long history of over-the-counter use as a remedy for many ailments, whereas TMAO is produced by gut microbes and enters the circulatory system, particularly following high-protein meals (Mitchell *et al.* 2019). Hence, these two molecules also have the potential to influence cellular function in humans, whether administered intentionally or otherwise. More broadly, proof-of-principle studies in *E. coli* make it abundantly clear that intracellular metabolites influence proteostasis and aid in mutational buffering (Verma *et al.* 2020) in a manner that depends on the biophysical effects of mutations (Bandyopadhyay *et al.* 2012; Dandage *et al.* 2018). Thus by modifying the chemical environment in which proteins fold and function and buffering fitness against the effects of mutations, naturally occurring small molecules have almost certainly influenced the evolution of protein sequence space.

## Data availability

Strains and plasmids are available upon request. The authors affirm that all data necessary for confirming the conclusions of the article are present within the article, figures, tables, and supplemental materials. Supplementary files are available at figshare: https://doi.org/10.25387/g3.14773530. Supplementary Table S1 has information about the TS alleles of essential genes in the “Boone TS collection” that we screened. Supplementary Table S2 has information about the mutants from the Boone TS collection that were rescued by, or sensitive to, TMAO. Supplementary Figure S1 provides the sequence of “recoded” *URA3* genes used for CRISPR-Cas9-based integration into the yeast genome. Supplementary Figure S2 has data regarding the dispensability of Arg 599 for *E. coli* β-gal activity *in vivo*. Supplementary Figure S3 defines room temperature in our experiments. Supplementary Figure S4 provides data regarding GdnHCl sensitivity in WT, TS, and *erg6*Δ mutants. Supplementary Figures S5 and S6 contain data showing the failure of urea to rescue TS mutants. Supplementary Figure S7 has data showing that DMSO or TMAO cannot replace cytosolic Hsp70 function. Supplementary Figure S8 contains data regarding the restrictive temperatures of TS mutants and how this relates to their ability to be rescued by TMAO. Supplementary Figure S9 shows examples of TMAO rescue of TS mutants.
